# USP21-mediated G3BP1 stabilization accelerates proliferation and metastasis of esophageal squamous cell carcinoma via activating Wnt/β-Catenin signaling

**DOI:** 10.1038/s41389-024-00524-3

**Published:** 2024-06-21

**Authors:** Jiazhong Guo, Yunpeng Zhao, Huacong Sui, Lei Liu, Fanrong Liu, Lingxiao Yang, Fengyuan Gao, Jinfu Wang, Yilin Zhu, Lingbing Li, Xiangqing Song, Peng Li, Zhongxian Tian, Peichao Li, Xiaogang Zhao

**Affiliations:** 1 Department of Critical Care Medicine, The Second Hospital, Cheeloo College of Medicine, Shandong University, Jinan, China; 2 Department of Thoracic Surgery, The Second Hospital, Cheeloo College of Medicine, Shandong University, Jinan, China; 3 Key Laboratory of Chest Cancer, The Second Hospital, Cheeloo College of Medicine, Shandong University, Jinan, China

**Keywords:** Oncogenes, Ubiquitylation

## Abstract

Lacking effective therapeutic targets heavily restricts the improvement of clinical prognosis for patients diagnosed with esophageal squamous cell carcinoma (ESCC). Ubiquitin Specific Peptidase 21 (USP21) is dysregulated in plenty of human cancers, however, its potential function and relevant molecular mechanisms in ESCC malignant progression as well as its value in clinical translation remain largely unknown. Here, in vitro and in vivo experiments revealed that aberrant upregulation of USP21 accelerated the proliferation and metastasis of ESCC in a deubiquitinase-dependent manner. Mechanistically, we found that USP21 binds to, deubiquitinates, and stabilizes the G3BP Stress Granule Assembly Factor 1 (G3BP1) protein, which is required for USP21-mediated ESCC progression. Further molecular studies demonstrated that the USP21/G3BP1 axis played a tumor-promoting role in ESCC progression by activating the Wnt/β-Catenin signaling pathway. Additionally, disulfiram (DSF), an inhibitor against USP21 deubiquitylation activity, markedly abolished the USP21-mediated stability of G3BP1 protein and significantly displayed an anti-tumor effect on USP21-driving ESCC progression. Finally, the regulatory axis of USP21/G3BP1 was demonstrated to be aberrantly activated in ESCC tumor tissues and closely associated with advanced clinical stages and unfavorable prognoses, which provides a promising therapeutic strategy targeting USP21/G3BP1 axis for ESCC patients.

## Introduction

Esophageal cancer, as a complex invasive disease, has become the seventh most common human malignancy and is the sixth leading cause of death related to human cancers worldwide [1]. There are two histological types of esophageal cancer, including esophageal squamous cell carcinoma (ESCC) and esophageal adenocarcinoma, which vary greatly in geographical distribution [2, 3]. Esophageal adenocarcinoma accounts for most of the esophageal cancer in Western countries, while 90% of yearly cases of esophageal cancer in Asia and sub-Saharan Africa were ESCC [3]. The incidence of esophageal cancer in China is higher than in any other country, which accounts for about 50% of the diagnosed cases of esophageal cancer worldwide [4, 5]. Although surgical treatment, neoadjuvant chemoradiotherapy, and immunotherapies have made significant progress in ESCC therapy, the 5-year survival rate of patients is only 36.9% in China and 18.5% in the United States from 2015 to 2017 [6]. Therefore, further exploring the molecular mechanism driving ESCC progression is extremely important in improving clinical prognosis.

Ubiquitination is very common in the post-translational modifications of proteins and plays crucial roles in protein stability and functions, which is tightly regulated by ubiquitin-activating (E1), ubiquitin-conjugating (E2), ubiquitin-ligating (E3) enzymes, and deubiquitinases (DUBs) [7–9]. Imbalanced ubiquitination induced by dysregulated E3 or DUBs impairs many cellular physiological processes, such as DNA damage repair, cell cycle, gene expression, and signaling transduction, which are closely associated with the initiation and progression of human cancers [10–12]. Our previous study clarified that ubiquitin-specific peptidase 36 (USP36) accelerates ESCC proliferation and metastasis through stabilizing YAP protein [13]. However, further studies are still needed to further elucidate the ambiguous mechanisms underlying dysregulated ubiquitination in ESCC progression.

Ubiquitin-specific peptidase 21 (USP21) is a member of the USP subfamily of DUBs. It has been reported that USP21 expression levels were dysregulated in a variety of human malignancies, including bladder cancer, breast cancer, colorectal cancer, gastric cancer, hepatocellular carcinoma, cholangiocarcinoma, lung cancer, pancreatic cancer, renal cell carcinoma, in which increased USP21 promoted the malignant phenotypes either by its activity of deubiquitinase to stabilize target proteins or via acting as a transcription factor to regulate the expression of target genes [14–25]. However, the exact roles of USP21 in the malignant progression of ESCC and its potential molecular mechanisms remain poorly understood.

In this study, we identified the oncogenic role of USP21 in ESCC proliferation and metastasis. Molecular mechanism exploration revealed that USP21 can bind to, deubiquitinize, and stabilize G3BP Stress Granule Assembly Factor 1 (G3BP1) protein to activate the Wnt/β-Catenin signaling which accelerates the malignant progression of ESCC. Interestingly, disulfiram (DSF), an inhibitor against the deubiquitylation activity of USP21, was demonstrated to possess obvious anti-cancer effects on the USP21-driving progression of ESCC. In conclusion, targeting USP21/G3BP1 could provide an effective therapeutic strategy for ESCC patients.

## Materials and methods

### Clinical ESCC samples

A total of 86 pairs of tissue samples were collected from ESCC patients undergoing surgery from 2016 to 2023 at The Second Hospital of Shandong University. Detailed clinical information was summarized in Supplementary Table 1. After surgical resection, all tissues were immediately fixed with formalin and then embedded with paraffin for immunohistochemical staining. The tumor-node-metastasis classification for the 86 cases of ESCC tumor tissues was performed according to the eighth edition of the American Joint Committee on Cancer and the Union for International Cancer Control [26]. Another 10 pairs of fresh ESCC samples were collected to detect USP21 mRNA levels. Informed consents were obtained from all patients. All procedures involved in the collection of ESCC samples and clinical information were approved and supervised by the Ethics Committee of The Second Hospital of Shandong University (KYLL-2022P231).

### Immunohistochemistry (IHC) analysis

Paraffin-embedded tissues were cut into sections with 4 µm thick and then subjected to dewaxing, rehydration, antigen retrieval, and blockage of endogenous peroxidase. After blocking nonspecific binding with goat serum at room temperature for 30 min, a primary antibody against the indicated protein was applied to incubate the sections at 4 °C overnight. Then, the sections were incubated with a secondary antibody conjugated by horseradish peroxidase at room temperature for 30 min followed by being developed signal with DAB solution (ORIGENE, ZLI-9018). Finally, the sections were counterstained with hematoxylin followed by dehydration and sealing. The images of IHC were obtained by using a NanoZoomer S60 Digital Pathology slide scanner (HAMAMATSU, Japan). IHC scores from each section were calculated by multiplying positive cell ratio (0, 0%; 1, 1–25%; 2, 26–50%; 3, 51–75%; 4, 76–100%) and staining intensity (0, negative; 1, weak; 2, moderate; 3, strong) [27]. Mean values of IHC scores from the 86 cases of tumor tissues were selected as the criteria for grouping. High USP21 or G3BP1 protein levels were defined when IHC scores were more than the mean, while low protein levels indicated their IHC scores were less than the mean values. The information on antibodies used in IHC was summarized in Supplementary Table 2.

### Cell culture

Human esophageal epithelial cells (HEEC) were purchased from the BeNa Culture Collection (Beijing, China). The human ESCC cell lines (Eca-109, KYSE-150, KYSE-410, and KYSE-510) and HEK293T cells were obtained from FuHeng Biology (Shanghai, China). Another ESCC cell line (KYSE-30) was purchased from CELLCOOK (Guangzhou, China). Before initiating cell experiments, Short Tandem Repeat (STR) analysis (Tsingke Biotechnology Co., Ltd., China) was applied to authenticate all cell lines. Eca-109, KYSE-150, KYSE-30, KYSE-410, and KYSE-510 cells were cultured with RPMI1640 medium (Corning, 10-040-CVRC) supplemented with 10% fetal bovine serum (FBS) (ExCell Bio, FSP500) and 1% Penicillin-Streptomycin (PS) (New Cell & Molecular Biotech, C125C5) while HEEC and HEK293T cells were maintained in DMEM medium (Corning, 10-013-CVRC) containing 10% FBS and 1% PS. All cells grow in a humidified incubator under the conditions of 37 °C and 5% CO_2_.

### Cell transfection

A total of 200,000 cells were seeded into each well of a six-well plate. When cell confluence reached about 70%, indicated plasmids or siRNAs were transfected into cells by applying Lipofectamine 3000 (Thermo Fisher Scientific, L3000015) or Lipofectamine RNAi MAX (Thermo Fisher Scientific, 13778150) following the manufacturer’s protocols. After 48 h of transfection, cells were harvested for the extraction of protein or RNA. Detailed plasmid information and siRNA sequence are listed in Supplementary Tables 3 and 4.

### Establishment of stable cell lines

The lentivirus was produced by transfecting two lentiviral packaging plasmids (pMD2.G and psPAX2) and the plasmids expressing target genes or shRNAs into HEK293T cells via Lipo293^TM^ Transfection Reagent (Beyotime, C0521). Then, ESCC cell lines were infected with the indicated lentivirus followed by being screened with a culture medium containing 1 μg/mL puromycin (Solarbio, P8230). Detailed information on used plasmids is present in Supplementary Table 3.

### Western blot analysis

Experimental cells were collected for protein extraction by using a boiling buffer consisting of 1% SDS, 10 mM Tris-HCl (pH 7.4), and 1 mM Na_3_VO_4_. SDS-PAGE was applied to separate proteins with different molecular weights. Then, the separated proteins were transferred onto nitrocellulose membranes (Merck Millipore, HATF00010). To block nonspecific binding, tris-buffered saline (TBS) containing 5% non-fat milk was used to incubate membranes for an hour at room temperature. The membranes were incubated with indicated primary antibody overnight at 4 °C followed by being incubated with secondary antibodies at room temperature for an hour. Finally, the signals from the targeted protein were detected in the Tanon Imaging System (Tanon) by using an enhanced chemiluminescent substrate (Boster, AR1197). The information on all antibodies used in the western blot was summarized in Supplementary Table 2. The full and uncropped images for western blots were uploaded to the Supplementary data. The bands from three replicate experiments were quantified using Image J software [28].

### Reverse transcriptional and quantitative polymerase chain reaction (RT-qPCR)

RNAsimple Total RNA Kit (Tiangen, DP419) was used to extract total RNA from experimental cells according to the manufacturer’s instructions. Reverse transcription was performed using LunaScript™ RT SuperMix Kit (NEB, E3010) while qPCR was conducted in a QuantStudioTM 5 System (Thermo Fisher Scientific) by applying Power SYBR® Green Master Mix (Thermo Fisher Scientific, 4367659). A detailed description of RT-qPCR conditions and results analysis was present in our previous work [29]. The sequences of primers are listed in Supplementary Table 5. ACTB or GAPDH mRNA level was used as an internal control.

### Co-immunoprecipitation (Co-IP)

Experimental cells were lysed by applying a Cell Lysis Buffer (CST, 9803). To remove nonspecific binding, cell lysates were incubated with IgG and Protein A Agarose (Beyotime, P2006) in an agitator for an hour at 4 °C. Then, centrifugation was performed to remove agarose beads and collect cleared lysates. 10% cleared lysates were preserved for input control while the indicated antibody was added to the remaining cleared lysates followed by constant agitation overnight at 4 °C. Next, Protein A Agarose was mixed with the above lysates at 4 °C for another 2 h of agitation. After washing and centrifugation, agarose beads were collected and resuspended with a 5× loading buffer. The protein complex from agarose beads was heated for 10 min at 100 °C and then subjected to SDS-PAGE and immunoblotting. The antibodies used in Co-IP are listed in Supplementary Table 2.

### Polyubiquitination detection assay

The ubiquitination levels of the G3BP1 protein were detected in HEK293T cells. HA-G3BP1- and His-Ubiquitin-expressing plasmids combined with another plasmid-expressing vector, Flag-USP21^WT^, or Flag-USP21^C221A^ were transfected into HEK293T cells using Lipofectamine 3000 (Thermo Fisher Scientific, L3000015). After 48 h of transfection, 20 μM of MG132, a proteasome inhibitor, (MCE, HY-13259) was applied to treat cells for 6 h. Cell lysate from transfected cells was obtained using lysis buffer (Beyotime, P0013) and then precleared through Protein A/G Magnetic Beads (MCE, HY-K0202) and IgG for 2 h. Co-IP with anti-HA-tag Magnetic Beads (MCE, HY-K0201) was performed to collect the complex binding to HA-G3BP1, and immunoblotting was applied to detect the indicated proteins. Detailed information about antibodies and plasmids used here are listed in Supplementary Tables 2 and 3.

### Dual-luciferase reporter assay

The transcription activity induced by β-Catenin was evaluated by using dual-luciferase reporter assays. TOPFlash or FOPFlash plasmids expressing firefly luciferase (Beyotime) combined with pRL-TK plasmids expressing renilla luciferase (Tsingke Biotechnology) were transfected into KYSE-150 and Eca-109 cells using Lipofectamine® 3000 Transfection Kit (Invitrogen, L3000-015) for 48 h. The luciferase activity from transfected cells was measured by the Dual-luciferase® Reporter Assay System (Promega, E1960) on an Automated Imaging Microplate Reader (Biotek, USA). FOPFlash was used as the negative control for TOPFlash. The renilla luciferase activities were used as an internal control for cell viability, transfection efficiency, and cell lysis. The transcription activity of Wnt/β-Catenin signaling was assessed by calculating the ratio of firefly to renilla luciferase activities. The plasmids used here are present in Supplementary Table 3.

### Plate colony formation assay

A total of 1000 experimental cells were cultured in each well of a 6-well plate. After 2 weeks of growth in an incubator with 5% CO_2_ at 37 °C, cell colonies were fixed with 4% paraformaldehyde for 15 min at room temperature followed by being stained with 0.1% crystal violet for 30 min. The ability of plate colony formation was evaluated according to the formula: (the number of colonies/1000) × 100%.

### Transwell assay

Experimental cells were plated in transwell chambers (Corning, 3422) to evaluate cell migration. BD Matrigel Matrix (BD Biosciences, 356234) was precoated on transwell chambers for cell invasion assay. In brief, the chamber was placed in one well containing 600 μL complete medium in a 24-well plate. Then, 40,000 cells suspended in medium without FBS were plated on transwell chambers for 48 h of growth in an incubator with 5% CO_2_ at 37 °C. Cells on chambers were fixed with 4% paraformaldehyde for 15 min at room temperature. After slightly scraping non-migrating or non-invading cells off, an inverted microscope and MShot Image Analysis System (Mshot, Guangzhou, China) were applied to quantify and photograph the migrating or invading cells.

### Xenograft model in vivo

All experiments about mice in this study were approved and supervised by the Ethical Committee for Animal Experimentation of The Second Hospital of Shandong University (KYLL-2022A195) and performed in the Animal Research Center of The Second Hospital of Shandong University. All BALB/c athymic nude mice (4 weeks old, male) were purchased from Beijing Vital River Laboratory Animal Technology Co., Ltd. (Beijing, China) and raised in a sterile condition with a 12-h light/dark cycle. To assess the ability of cell proliferation in vivo, 100 μL PBS containing 5 × 10^6^ cells was subcutaneously xenografted in the axillary fossa of each mouse. The volume of xenograft tumor was evaluated every 3 days and calculated according to the formula (longest-diameter × shortest-diameter^2^/2). Once the maximum size of the tumor reached or exceeded 1 cm^3^, experiments were terminated, and all mice were euthanized. For the metastasis model in vivo, 1 × 10^6^ cells suspended in 100 μL PBS were injected into each mouse through the tail vein. All mice were sacrificed after 6 weeks, and the lung was excised for evaluation of metastatic nodules. After being fixed with formalin and embedded with paraffin, all xenograft tumors and mice lungs were cut into sections with 4 μm thick for IHC and H&E staining.

### Statistical analysis

GraphPad Prism version 8.0.0 for Windows (GraphPad Software, San Diego, California, USA) was applied to analyze and visualize all data from at least three independent experiments. Non-parametric (Wilcoxon matched-pairs signed rank test and Mann-Whitney test) or parametric statistical analysis (paired *t-*test, unpaired *t-*test, and two-way ANOVA) was applied to determine significant differences between the indicated two groups. The prognostic value of USP21 and G3BP1 expression levels was assessed with Kaplan-Meier analysis by using the log-rank test. IBM SPSS Statistics for Windows, version 25 (IBM Corp., Armonk, New York, USA) was applied to establish the Cox-regression Hazard models. The correlation analysis between USP21 and G3BP1 protein levels in ESCC samples was performed by the Spearman correlation coefficient. When *P* values less than 0.05, differences possess statistical significance.

## Results

### USP21 expression is aberrantly upregulated and indicates an unfavorable prognosis in ESCC

**Fig. 1:**
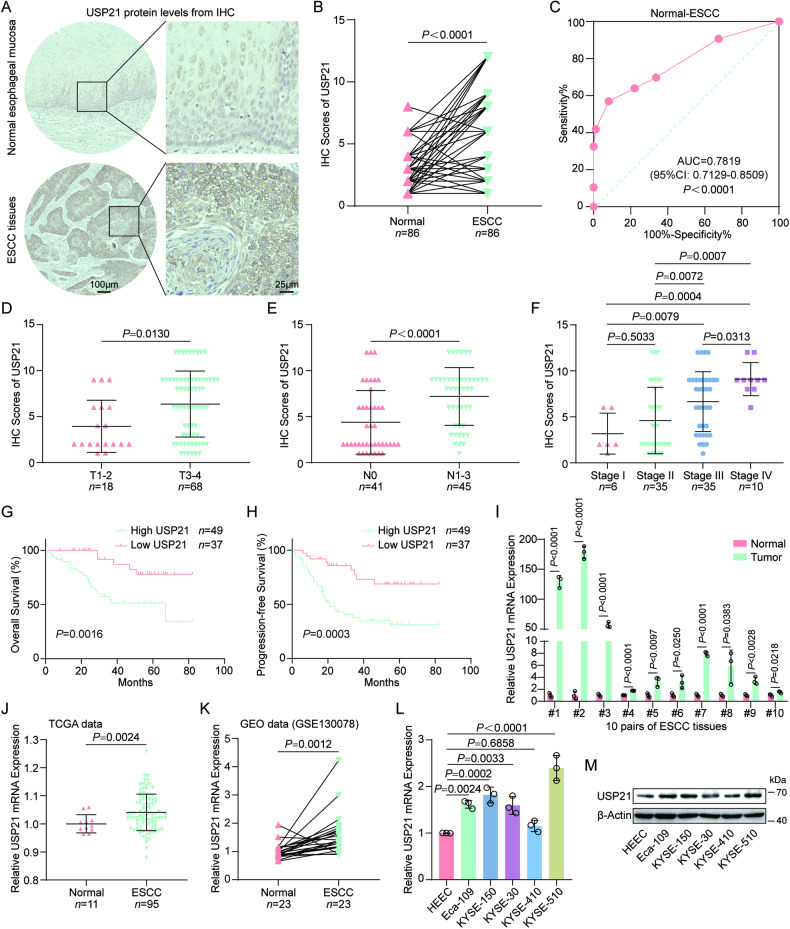
USP21 expression is aberrantly upregulated and indicates an unfavorable prognosis in ESCC. **A**, **B** USP21 protein levels in ESCC tumor and adjacent normal esophageal mucosa tissues were evaluated by IHC. Representative staining images with scale bars (**A**) and a comparison of IHC scores (**B**) are present. **C** The diagnostic value of USP21 protein levels for ESCC was evaluated using the receiver operating characteristic (ROC) analysis. Further analysis based on IHC scores from ESCC samples with different primary tumor statuses (T1-2 and T3-4) (**D**), with no metastasis (N0) or metastasis (N1-3) of regional lymph nodes (**E**), with different stages (I, II, III, and IV) (**F**). Prognostic differences in patients with high or low levels of USP21 were assessed by Kaplan-Meier curves for overall survival (OS) (**G**) and progression-free survival (PFS) (**H**). **I** USP21 mRNA levels were detected in 10 pairs of fresh ESCC tissues through RT-qPCR. The expression of USP21 mRNA was evaluated using TCGA data from the UCSC Xena platform (**J**) and GEO data (GSE130078) (**K**). USP21 mRNA (**L**) and protein levels (**M**) were detected using RT-qPCR and western blot in HEEC, Eca-109, KYSE-150, KYSE-30, KYSE-410, and KYSE-510 cells. Statistical significance was determined by applying a Wilcoxon matched-pairs signed rank test (**B**), a Mann-Whitney test (**D**–**F**), log-rank test (**G**, **H**), unpaired *t*-test (**I**, **J**, **L**), or paired *t*-test (**K**). All *P* values and n-numbers are indicated on the corresponding graphs.

Although one recent study reported increased protein levels of USP21 in 4 cases of esophageal cancer tissues [30], USP21 expression levels in ESCC (the most predominant pathological subtype of esophageal cancer in Asian populations), its association with clinicopathological factors, and its effect on ESCC prognosis remain unknown. Here, we detected USP21 protein levels in 86 pairs of ESCC samples with complete clinical information (Supplementary Table 1). IHC scores demonstrated that ESCC tissues had significantly upregulated levels of USP21 protein compared to adjacent normal esophageal mucosa (Fig. 1A, B). Receiver operating characteristic (ROC) analysis revealed that USP21 protein levels possess the potential ability to distinguish ESCC tumors from their matched normal tissues (Fig. 1C). Further analysis based on IHC evaluation exhibited that ESCC tumor tissues at advanced statuses of primary tumor (T3-4) or with regional lymph node metastasis (N1-3) had higher levels of USP21 protein compared with those at early primary tumor statuses (T1-2) (Fig. 1D) or without positive lymph nodes (N0) (Fig. 1E). Consistently, USP21 protein expression in stage III or IV ESCC specimens was obviously higher than that in ESCC tissues with stage I and II or with stage I, II, and III (Fig. 1F). Additionally, poorly differentiated (G3) ESCC tumor samples displayed increased USP21 protein levels compared to well/moderately differentiated (G1-2) tumors (Fig. S1A). However, no significant association was found between USP21 protein levels and other clinical characteristics (age and gender) (Fig. S1B, C). To determine the effect of USP21 protein levels on ESCC prognosis, 86 patients were categorized into low or high USP21 expression groups according to IHC scores. Kaplan-Meier analyses demonstrated that ESCC patients with high USP21 levels displayed a shorter overall survival (OS) and progression-free survival (PFS) than patients with low expression of USP21 (Fig. 1G, H). To clarify whether USP21 protein expression is an independent predictor for the prognosis of ESCC patients, we performed a multivariate analysis by establishing the Cox-regression Hazard models. As shown in Supplementary Table 6, the status of the regional lymph node (N1-3 vs. N0) can independently predict OS and PFS, while USP21 protein expression (High vs. Low) is only an independent predictor for PFS. These results suggest that in terms of prognostic prediction for ESCC patients, USP21 may be no more useful than the standard staging, especially compared with the status of regional lymph nodes. Additionally, USP21 mRNA levels in 10 cases of fresh ESCC tumor samples were obviously upregulated compared with adjacent normal tissues (Fig. 1I). Consistently, increased mRNA levels of USP21 in ESCC tissues were identified based on TCGA data from the UCSC Xena platform [31] and GEO data (GSE130078) [32] (Fig. 1J, K). USP21 expression was further evaluated in normal human esophageal epithelial cells (HEEC) and five ESCC cell lines (Eca-109, KYSE-150, KYSE-30, KYSE-410, and KYSE-510). As shown in Fig. 1L, M, USP21 mRNA and protein levels in four ESCC cell lines (Eca-109, KYSE-150, KYSE-30, and KYSE-510) were remarkedly elevated compared to those in HEEC cells. Collectively, our results suggest dysregulated expression of USP21 in ESCC, which predicts a worse clinical prognosis.

### Dysregulated USP21 accelerates ESCC progression through its deubiquitinase activity in vitro and in vivo

**Fig. 2:**
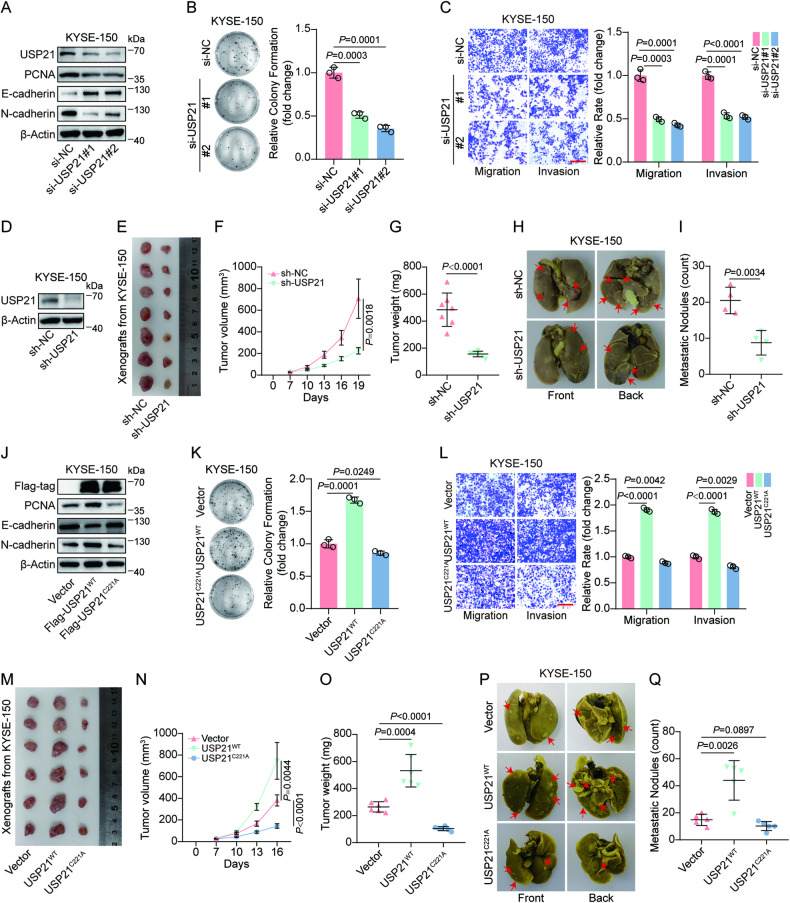
Dysregulated USP21 accelerates ESCC progression through its deubiquitinase activity in vitro and in vivo*.* **A** KYSE-150 cells were transfected with si-NC, si-USP21#1, or si-USP21#2. Western blot analysis was used to detect the protein levels of USP21, PCNA, E-Cadherin, and N-Cadherin. Plate colony formation assays (**B**) were applied to assess the proliferation ability of KYSE-150 cells transfected with si-NC, si-USP21#1, or si-USP21#2, while transwell assays (**C**) were used to evaluate cell migration and invasion capability. Representative staining and quantification as indicated. **D** Western blot analysis for verifying USP21 protein levels in stable KYSE-150 cells with sh-USP21 or sh-NC expression. The image of xenografts (**E**), tumor volume curve (**F**), and quantification for tumor weights (**G**) from BALB/c-nude mice (*n* = 7) subcutaneously xenografted with KYSE-150 cells expressing sh-USP21 or sh-NC for 19 days. **H**, **I** KYSE-150 cells with sh-USP21 or sh-NC expression were injected into the tail vein of BALB/c-nude mice (*n* = 4) for 6 weeks. Representative images (**H**) and statistical analysis (**I**) of metastatic nodules in mice lungs. **J** Western blot analysis was applied to determine the indicated protein levels in KYSE-150 cells with Vector, Flag-USP21^WT^, or Flag-USP21^C221A^ expression. The Vector-, USP21^WT^-, or USP21^C221A^-expressing KYSE-150 cells growth, migration, and invasion were evaluated by plate colony formation (**K**) and transwell assays (**L**). Representative images and quantification as shown. **M**–**O** KYSE-150 cells with Vector-, USP21^WT^-, or USP21^C221A^-expression were subcutaneously xenografted into BALB/c-nude mice (*n* = 6) for 16 days. The representative xenograft image (**M**), volume curve for tumor growth (**N**), and statistical analysis for tumor weights (**O**) as indicated. **P**, **Q** The Vector-, USP21^WT^-, or USP21^C221A^-expressing KYSE-150 cells were injected into BALB/c-nude mice (*n* = 5) through a tail vein for 6 weeks. Representative images (**P**) and statistical analysis (**Q**) for pulmonary metastatic nodules as shown. Scale bars (red line) in (**C**) and (**L**) are 100 μm. The data in (**B**, **C**, **F**, **G**, **I**, **K**, **L**, **N**, **O**, **Q**) are presented as means ± SD. An unpaired *t*-test was performed to determine the statistical significance in (**B**, **C**, **G**, **I**, **K**, **L**, **O**, **Q**) while a two-way ANOVA was used for (**F**, **N**). The *P* values are displayed in the corresponding panels, respectively.

In order to investigate the function of USP21 on ESCC progression, we knocked down endogenous USP21 expression in KYSE-150 and Eca-109 cells by transfecting siRNAs. The efficiency of USP21 depletion was verified by western blot and quantitation (Fig. 2A, Fig. S2A–C). To prove the specificity of siRNAs against USP21, we constructed the plasmid expressing siRNA-resistant USP21 (USP21^SR^) that translates the same sequence of amino acids (aa) as wild-type USP21 (USP21^WT^) but possesses mutant base sequence with resistance to si-USP21#1 and si-USP21#2. Our results revealed that si-USP21#1 or si-USP21#2 obviously reduced USP21^WT^ protein levels but did not affect USP21^SR^ expression (Fig. S2D). Functional experiments in vitro revealed that siRNA-regulated depletion of endogenous USP21 remarkedly suppressed plate colony formation (Fig. 2B, Fig. S2E), migration, and invasion (Fig. 2C, Fig. S2F) of KYSE-150 and Eca-109 cells. Consistently, USP21 knockdown significantly downregulated the protein levels of PCNA (a marker for cell proliferation) and N-Cadherin (a marker for mesenchymal cells) but upregulated the expression of E-Cadherin (a marker for epithelial cells) (Fig. 2A, Fig. S2B). To further demonstrate the consequence induced by USP21 depletion in vivo, we established ESCC cell lines with stably shRNA-mediated depletion of endogenous USP21 (sh-USP21) or negative control (sh-NC), in which stable USP21 knockdown efficiency was shown in Fig. 2D. In xenograft and lung metastasis mice models, we found that USP21 downregulation obviously inhibited tumor growth rate, weight and Ki-67 protein expression (Fig. 2E–G, Fig. S3A) and decreased metastatic nodules in mice lung (Fig. 2H, I, Fig. S3B). These results indicate the cancer-promoting function of USP21 in ESCC progression.

To determine whether the oncogenic role of USP21 in ESCC is dependent on its deubiquitination activity, we constructed plasmids expressing USP21^WT^ or mutated USP21 with catalytic inactivity (USP21^C221A^). As shown in Fig. 2J and Fig. S4A, USP21^WT^, and USP21^C221A^ expression levels were comparable in KYSE-150 and Eca-109 cells, which eliminated the effect of USP21 expression differences on subsequent functional experiments. Plate colony formation assays revealed that ectopic USP21^WT^ expression enhanced colony formation in KYSE-150 and Eca-109 cells compared with vector control, while USP21^C221A^ overexpression failed to promote the rates of colony formation (Fig. 2K, Fig. S4B). Similarly, in transwell assays, USP21^C221A^ displayed no tumor-facilitating function in cell migration and invasion compared to USP21^WT^ overexpression (Fig. 2L, Fig. S4C). Consistently, USP21^WT^ overexpression significantly increased the expression of PCNA and N-Cadherin but reduced E-Cadherin levels while ectopic USP21^C221A^ expression could not result in a similar alteration of these markers (Fig. 2J, Fig. S4A). The quantitation of western blots for PCNA, E-Cadherin, and N-Cadherin in KYSE-150 cells expressing Vector, USP21^WT^, or USP21^C221A^ is displayed in Fig. S4D–F. Furthermore, USP21^WT^, but not USP21^C221A^ mutation, significantly promoted tumor growth and upregulated Ki-67 protein levels in subcutaneous xenograft models (Fig. 2M–O, Fig. S5A) and increased the number of metastatic nodules in experimental mice models with lung metastasis (Fig. 2P, Q, Fig. S5B). Interestingly, USP21^C221A^ displayed an inhibition on xenograft tumor growth compared with vector control in vivo (Fig. 2M–O). In summary, our results suggest the oncogenic function of USP21 in ESCC is exactly dependent on its activity of deubiquitinase.

### USP21 binds to, deubiquitinates, and stabilizes the G3BP1 protein

**Fig. 3:**
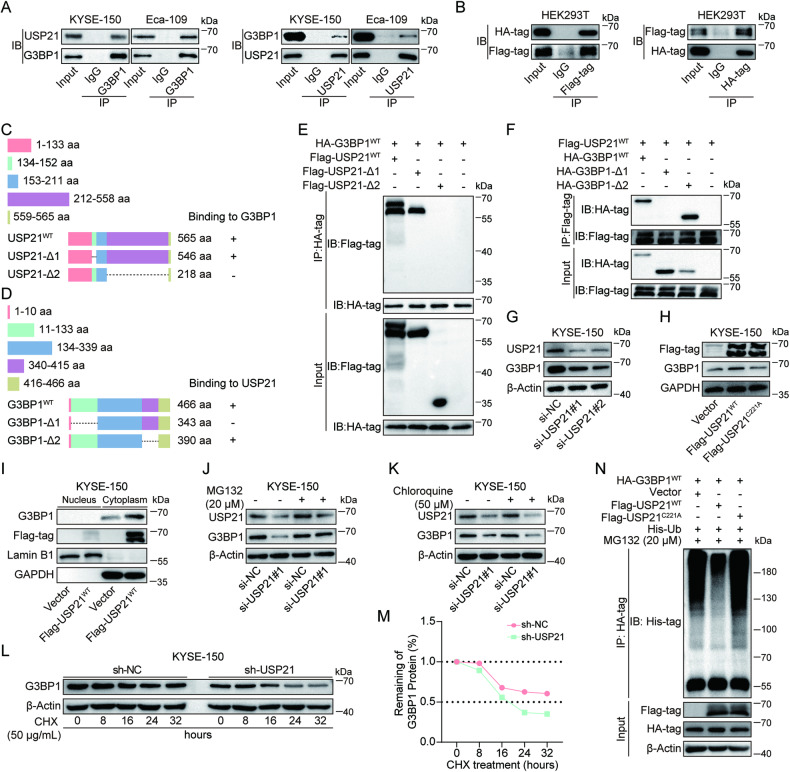
USP21 binds to, deubiquitinates, and stabilizes G3BP1. **A** Co-immunoprecipitation (Co-IP) assays were applied to validate the interaction between endogenous USP21 and G3BP1 proteins in KYSE-150 and Eca-109 cells using antibodies against G3BP1 or USP21. **B** HEK293T cells were transfected with Flag-USP21^WT^- and HA-G3BP1^WT^-expressing plasmids. Co-IP assays with Flag or HA antibody were used to determine the binding of ectopic USP21 and G3BP1. **C**, **D** Schematic diagram displaying wild type or truncated mutation of USP21 and G3BP1 proteins. Co-IP assays with HA or Flag antibody were applied to determine the bindings between truncated USP21 and G3BP1^WT^ (**E**) or between USP21^WT^ and truncated G3BP1 proteins (**F**) followed by immunoblotting with indicated antibodies. Western blot analysis was performed to detect G3BP1 protein levels in KYSE-150 cells with USP21 depletion (**G**) or overexpression (**H**). **I** The nucleus and cytoplasmic proteins were extracted separately for western blot with G3BP1 protein in KYSE-150 cells with ectopic USP21^WT^ expression. GAPDH was used as an internal control for cytoplasmic lysate while Lamin B1 was applied for the loading control of nucleus proteins. KYSE-150 cells were transfected with si-NC or si-USP21#1 followed by treatment with Vehicle, MG132 (20 μM) (**J**), or chloroquine (50 μM) (**K**) for 24 h. Western blot analysis was used for the detection of G3BP1 protein levels. **L**, **M** KYSE-150 cells with stable expression of sh-NC or sh-USP21 were treated with 50 μg/mL of Cycloheximide (CHX), a protein synthesis inhibitor, for 0, 8, 16, 24, and 32 h. Western blot analysis (**L**) and quantification of protein half-life (**M**) for G3BP1 protein as shown. **N** HEK293T cells were transfected with G3BP1^WT^-, Vector/USP21^WT^/USP21^C221A^-, and Ub-expressing plasmids followed by MG132 (20 μM) treatment for 6 h. Co-IP assays were applied to detect G3BP1 ubiquitination.

It has been reported that USP21 acts as a deubiquitinase to stabilize certain oncoproteins, which promotes the progression of many human cancers [14, 15, 17, 19, 20, 23, 24]. Hence, to find the potential target regulated by USP21 in ESCC, we performed Co-IP assays to obtain a USP21-containing complex in KYSE-150 cells with ectopic expression of Flag-USP21^WT^, followed by mass spectrometry analysis (data not shown). Among all potential proteins binding to USP21, G3BP1 aroused our attention and interest, because G3BP1 displayed a relatively high enrichment in the Co-IP complex and was reported as an oncogene in ESCC progression [33]. The physical binding between USP21 and G3BP1 proteins was further verified in KYSE-150 and Eca-109 cells by using an antibody against G3BP1 or USP21 (Fig. 3A). In addition, we determined the binding between ectopic USP21 and G3BP1 proteins in HEK293T cells through IP with Flag- or HA-antibody (Fig. 3B). Subsequently, to map the essential domains mediating the binding between USP21 and G3BP1 protein, we constructed truncated USP21- or G3BP1-expressing plasmids, Flag-USP21-$\Delta$1 with the deletion of nuclear export signal (NES) domain (134-152 aa), Flag-USP21-$\Delta$ 2 with the deletion of USP domain (212-558 aa), HA-G3BP1-$\Delta$ 1 lacking the domain of nuclear transport factor 2 (NTF2) (11-133 aa), and HA-G3BP1-$\Delta$ 2 lacking the domain of RNA recognition motif (RRM) (340-415 aa) (Fig. 3C, D). As shown in Fig. 3E, F, the deletion of the USP domain (212-558 aa) in USP21 or the domain of NTF2 (11-133 aa) in G3BP1 abolished their binding, which suggests the indispensable role of the two domains in their physical interaction.

To determine USP21-mediated G3BP1 expression levels, we knocked down USP21 expression by applying siRNA in KYSE-150 and Eca-109, in which USP21 depletion downregulated the expression of G3BP1 protein but had no effect on G3BP1 mRNA levels (Fig. 3G, Fig. S6A, B). Furthermore, we explored how ectopic USP21^WT^- or USP21^C221A^-expression mediates G3BP1 protein levels. As expected, ectopic USP21^WT^ significantly increased G3BP1 protein expression compared with vector control, whereas USP21^C221A^ overexpression failed to upregulate the protein levels of G3BP1 (Fig. 3H, Fig. S6C). Neither USP21^WT^ nor USP21^C221A^ expression altered G3BP1 mRNA levels (Fig. S6D). In addition, we found that USP21-mediated upregulation of G3BP1 predominantly occurred in the cytoplasm (Fig. 3I, Fig. S6E).

Interestingly, reduced G3BP1 protein induced by USP21 depletion was abolished by the treatment of MG132, a proteasome inhibitor, but not chloroquine, an autophagy inhibitor (Fig. 3J, K, Fig. S7A, B). These results indicate that USP21 mediates G3BP1 protein levels via proteasome-dependent degradation. Moreover, a time-dependent decrease of G3BP1 protein was found in KYSE-150 cells treated with cycloheximide (CHX), a protein synthesis inhibitor, however, USP21 knockdown markedly shortened the half-life of G3BP1 protein (Fig. 3L, M). To further verify the effect of USP21 deubiquitinase activity on G3BP1 protein levels, the plasmids of His-Ub and HA-G3BP1^WT^ combined with Flag-USP21^WT^- or Flag-USP21^C221A^-expressing plasmids were transfected into HEK293T cells, followed by MG132 treatment, in which USP21^WT^, but not USP21^C221A^, decreased the ubiquitination of G3BP1 protein compared with vector control (Fig. 3N). Collectively, our results strongly demonstrate that USP21 enhances the stabilization of G3BP1 protein by binding to and deubiquitinating it.

### G3BP1 is essential for USP21-mediated ESCC progression

**Fig. 4:**
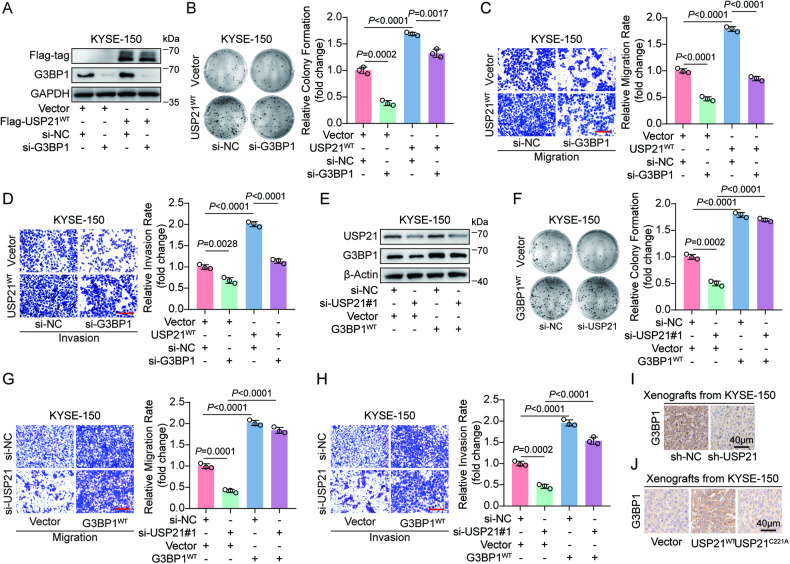
G3BP1 is essential for USP21-mediated ESCC progression. **A** Western blot analysis was conducted to determine indicated protein expression in Vector- or USP21^WT^-expressing KYSE-150 cells transfected with si-NC or si-G3BP1. Plate colony formation (**B**) and transwell assays (**C**, **D**) were performed to assess the growth, migration, and invasion of KYSE-150 cells expressing Vector + si-NC, USP21^WT^ + si-NC, Vector + si-G3BP1, or USP21^WT^ + si-G3BP1. Representative images and statistical quantification as indicated. **E** Western blot analysis was performed to detect indicated protein levels in Vector- or G3BP1^WT^-expressing KYSE-150 cells with transfection of si-NC or si-USP21#1. Plate colony formation (**F**) and transwell assays (**G**, **H**) were used to evaluate the growth, migration, and invasion of KYSE-150 cells expressing si-NC + Vector, si-NC + G3BP1^WT^, si-USP21#1 + Vector, or si-USP21#1 + G3BP1^WT^. Representative images and statistical quantification as shown. IHC analysis was performed to evaluate G3BP1 protein levels in the sections of mice xenograft tumor from the group of sh-NC/sh-USP21 (**I**) or Vector/USP21^WT^/USP21^C221A^ (**J**). Scale bars (red line) are 100 μm in (**C**, **D**, **G**, **H**). The data for statistical analysis are presented as means ± SD and the unpaired *t*-test was applied to determine the statistical significance (**B**–**D**, **F**–**H**). The *P* values are shown in the corresponding panels, respectively.

Considering that USP21 binds to and stabilizes G3BP1, we speculated that USP21 functions as an oncogene in ESCC via G3BP1. To demonstrate our hypothesis, KYSE-150 and Eca-109 cells were co-transfected with si-NC or si-G3BP1 combined with vector or USP21^WT^-expressing plasmids. The efficiency of ectopic USP21 expression and endogenous G3BP1 knockdown was verified in Fig. 4A and Fig. S8A. Plate formation and transwell assays revealed that USP21^WT^-induced enhancement of cell growth, migration, and invasion in KYSE-150 and Eca-109 cells was obviously abolished by G3BP1 depletion (Fig. 4B–D, Fig. S8B–D). To further identify the function of G3BP1 in USP21-mediated progression of ESCC, we transfected G3BP1-expressing plasmids to KYSE-150 and Eca-109 cells with siRNA-regulated USP21 knockdown, as shown in Fig. 4E and Fig. S8E. Cell function experiments in vitro demonstrated that weakened abilities of cell proliferation, migration, and invasion in KYSE-150 and Eca-109 cells with USP21 depletion were significantly restored by ectopic overexpression of G3BP1 (Fig. 4F–H, Fig. S8F–H). Additionally, IHC for mice xenograft sections revealed that G3BP1 protein levels in xenograft tumors were significantly decreased in USP21-depleted group (Fig. 4I). Consistently, G3BP1 protein was remarkedly increased in subcutaneous tumors derived from USP21^WT^-expressing, but not USP21^C221A^-expressing, KYSE-150 cells compared with the control group (Fig. 4J). Taken together, our results demonstrate that USP21 plays an oncogenic role in ESCC progression through upregulating G3BP1.

### USP21 activates Wnt/β-Catenin signaling to accelerate ESCC progression in a G3BP1-dependent manner

**Fig. 5:**
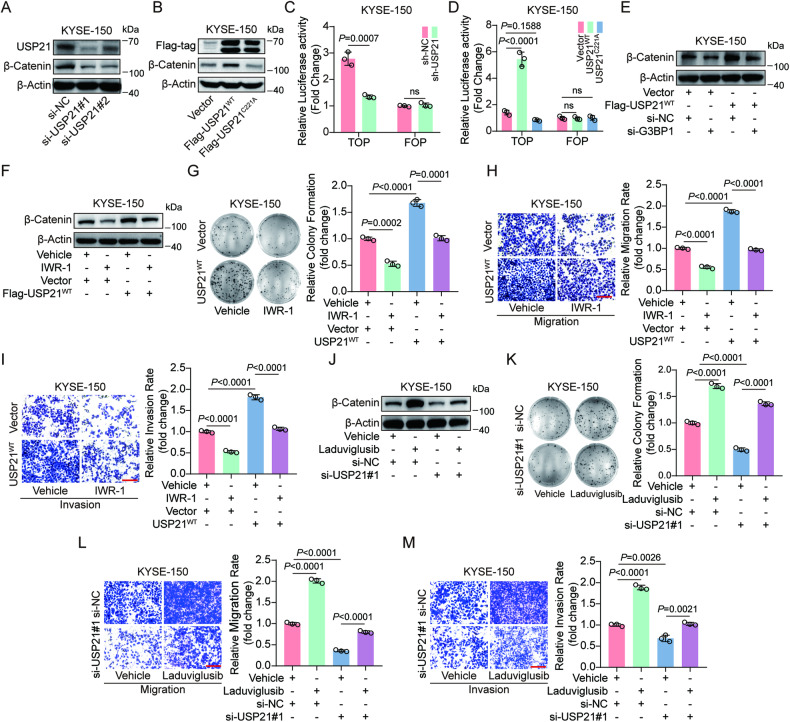
USP21 activates Wnt/β-Catenin signaling to accelerate ESCC progression in a G3BP1-dependent manner. Western blot analysis was used to detect β-Catenin protein levels in KYSE-150 cells transfected with si-NC, si-USP21#1, or si-USP21#2 (**A**) or transfected with Vector-, USP21^WT^-, or USP21^C221A^-expressing plasmids (**B**). Dual-luciferase reporter assays were used to measure the luciferase activity of TOP and FOP plasmids in KYSE-150 cells with stable USP21 knockdown (**C**) or overexpression (**D**). The pRL-TK plasmids were co-transfected as an internal control. **E** Western blot analysis was conducted to detect β-Catenin protein levels in KYSE-150 cells transfected with Vector + si-NC, USP21^WT^ + si-NC, Vector + si-G3BP1, or USP21^WT^ + si-G3BP1. **F**–**I** KYSE-150 cells expressing Vector or USP21^WT^ were treated with vehicle or 10 μM of IWR-1, an inhibitor for Wnt signaling, for 48 h. Western blot analysis for β-Catenin protein levels (**F**), plate colony formation for cell growth (**G**), and transwell assays for cell migration and invasion (**H**, **I**). **J**–**M** KYSE-150 cells were transfected with si-NC or si-USP21 followed by treatment with laduviglusib (10 μM), a potent activator for Wnt signaling, or vehicle for 48 h. Western blot to detect β-Catenin protein (**J**), plate colony formation to evaluate cell growth (**K**), and transwell assays to measure cell migration and invasion (**L**, **M**). Representative stainings and quantification as indicated. Scale bars (red line) are 100 μm in (**H**, **I**, **L**, **M**). Statistical significance was identified with an unpaired *t*-test, and all analyzed data are shown as means ± SD (**C**, **D**, **G**–**I, K**–**M**). The *P* values are respectively indicated in the corresponding position.

G3BP1 has been reported to regulate the activity of the Wnt/β-Catenin signaling pathway in esophageal cancer, colon cancer, and breast cancer [33–35]. In the present study, we found that knocking-down USP21 remarkedly reduced the expression level of β-Catenin, the key effector of Wnt/β-Catenin signaling, (Fig. 5A, Fig. S9A) while overexpression of USP21^WT^, but not USP21^C221A^, significantly upregulated β-Catenin levels (Fig. 5B, Fig. S9B). Given that increased accumulation of β-Catenin in the nucleus is a key indicator for the activation of Wnt signaling, we separately extracted the cytoplasmic and nuclear proteins from KYSE-150 transfected with si-NC, si-USP21#1 or si-USP21#2 or expressing Vector, USP21^WT^, or USP21^C221A^. Our results exhibited that the knockdown of USP21 obviously reduced nuclear accumulation of β-Catenin, while USP21^WT^, but not USP21^C221A^, increased β-Catenin protein levels in the cell nucleus (Fig. S9C, D). Furthermore, a dual-luciferase reporter assay revealed that the activity of luciferase from TOP plasmids was remarkedly inhibited by USP21 depletion but enhanced by the overexpression of USP21^WT^, but not USP21^C221A^, however, both USP21 knockdown and overexpression had no obvious effect on luciferase activity from FOP plasmids (Fig. 5C, D). These results solidly demonstrate the positive regulation of USP21 on Wnt/β-Catenin signaling. To figure out whether USP21-mediated activation of Wnt/β-Catenin signaling depends on G3BP1, we knocked down G3BP1 in USP21^WT^-expressing KYSE-150 cells and found that increased expression of β-Catenin induced by USP21 overexpression was obviously diminished by G3BP1 knockdown (Fig. 5E, S9E). Similarly, we overexpressed G3BP1 in USP21-depleted KYSE-150 cells and demonstrated that β-Catenin downregulation caused by USP21 depletion was significantly restored by ectopic G3BP1^WT^ expression (Fig. S9F). These results indicate that USP21 mediates the Wnt/β-Catenin signaling pathway in a G3BP1-dependent manner. Then, we asked whether USP21 promotes ESCC malignant biological behaviors via activating the Wnt/β-Catenin pathway. As shown in Fig. 5F and Fig. S9G, treatment with IWR-1, an inhibitor for Wnt/β-Catenin signaling, significantly decreased β-Catenin levels in USP21^WT^-expressing KYSE-150 cells. Plate colony formation and transwell assays revealed that enhanced proliferation, migration, and invasion of KYSE-150 cells with USP21^WT^ overexpression were significantly diminished by IWR-1 treatment (Fig. 5G–I). On the other hand, we found that laduviglusib, an activator for Wnt/β-Catenin signaling, obviously reversed the downregulation of β-Catenin protein (Fig. 5J, Fig. S9H) and rescued the weakened malignant phenotypes in KYSE-150 with USP21 depletion (Fig. 5K–M). Besides the experiments about the chemical modulator of the Wnt pathway, we introduced β-Catenin-expressing plasmid to genetically prove the role of the Wnt pathway in USP21-mediated ESCC progression. Our results revealed that ectopic β-Catenin expression significantly abolished si-USP21#1-induced inhibition of proliferation, migration, and invasion of KYSE-150 cells (Fig. S10A–D). In conclusion, our results demonstrate that USP21 exercises oncogenic effects in ESCC progression through regulating Wnt/β-Catenin.

### DSF inhibits USP21-drived ESCC progression

**Fig. 6:**
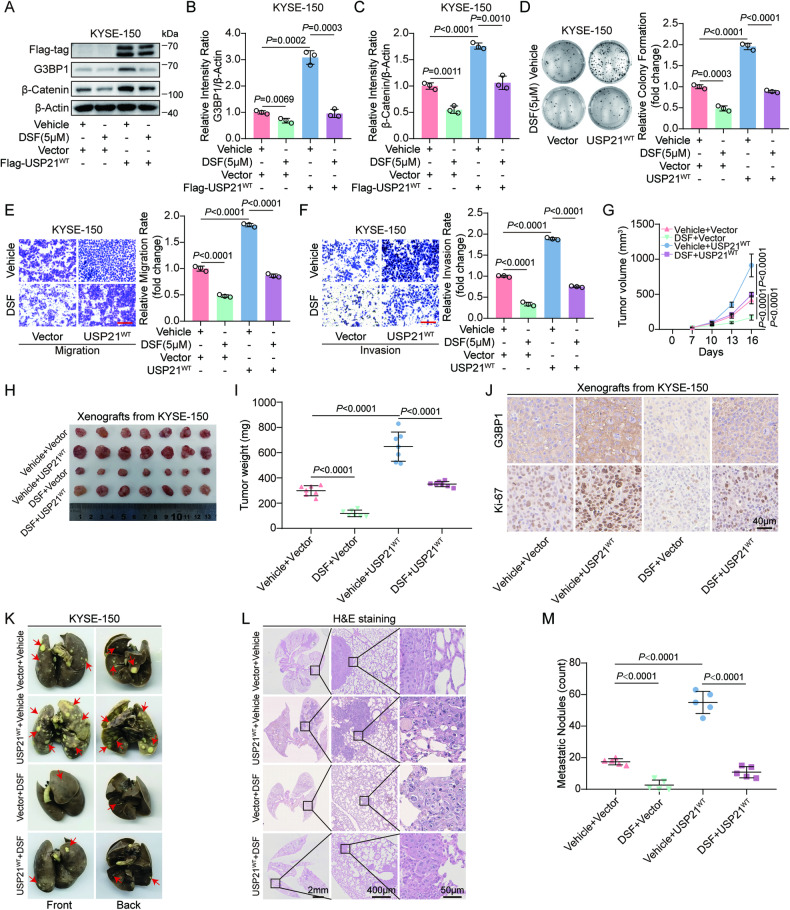
DSF inhibits the USP21-drived ESCC progression. **A**–**F** The vector- or USP21^WT^-expressing KYSE-150 cells were treated with 5 μM of DSF or vehicle control. Western blot analysis to detect G3BP1 and β-Catenin protein levels (**A**), quantitation for western blots of G3BP1 and β-Catenin from three replicate experiments as shown (**B**, **C**), plate colony formation to assess cell growth (**D**), and transwell assays to evaluate cell migration and invasion (**E**, **F**). Scale bars (red line) indicate 100 μm in (**E**, **F**). **G**–**I** BALB/c-nude mice (*n* = 7) were subcutaneously xenografted with vector- or USP21^WT^-expressing KYSE-150 cells for 16 days and treated with an intraperitoneal injection of DSF (200 mg/kg) or vehicle control every 2 days at the initiation of the model establishment. Tumor growth curve (**G**), the representative image of xenograft tumors (**H**), and tumor weights quantification (**I**) as indicated. **J** The protein levels of G3BP1 and Ki-67 in the sections of xenografts from (**H**) were evaluated using IHC. Scale bars as indicated. **K**–**M** KYSE-150 cells with vector or USP21^WT^ expression were injected into BALB/c-nude mice (*n* = 5) via tail vein for 6 weeks. At the initiation of establishing metastatic lung xenograft model, mice were treated with DSF (200 mg/kg) or vehicle control through an intraperitoneal injection every 2 days. Representative images for pulmonary metastatic nodules (**K**), corresponding H&E staining with the scale bars (**L**), and statistical analysis (**M**) as shown. All data for statistical analysis are presented as means ± SD. Statistical significance of differences was identified using an unpaired *t*-test for (**B**–**F**, **I**, **M**) or the two-way ANOVA for (**G**), and the *P* values are displayed correspondingly.

The anti-tumor effect of DSF has been demonstrated in many human cancers, such as breast cancer, lung cancer, pancreatic cancer, glioblastoma, melanoma, and esophageal cancer [36, 37]. Interestingly, it has been reported that DSF functions as a potent inhibitor against deubiquitinase activity of USP21 [38]. This prompted us to investigate whether DSF suppresses USP21-mediated G3BP1 upregulation, activation of Wnt/β-Catenin signaling, and ESCC malignant progression. We treated USP21^WT^-expressing KYSE-150 cells with DSF and found that USP21-induced upregulation of G3BP1 and β-Catenin protein was obviously diminished by DSF treatment according to Western blot analysis and its quantitation (Fig. 6A–C). Plate colony formation and transwell assays demonstrated that USP21-mediated enhancement of cell proliferation, migration, and invasion was remarkedly abolished in KYSE-150 cells when treated with DSF (Fig. 6D–F). Furthermore, in vivo experiments revealed that USP21^WT^ overexpression accelerated subcutaneous tumor growth of KYSE-150 cells in mice xenograft models, however, DSF administration significantly abolished USP21^WT^-induced tumor proliferation (Fig. 6G–I). Consistently, IHC for xenograft tumor section indicated that the protein levels of Ki-67 and G3BP1 in USP21^WT^-expressing group were obviously upregulated compared with those in vector control, while USP21^WT^-induced increase of these proteins was greatly diminished by DSF treatment (Fig. 6J). Similarly, in experimental lung metastasis models, USP21^WT^-expressing KYSE-150 cells formed more pulmonary nodules than vector control, while DSF significantly decreased the number of USP21^WT^-induced nodules in mice lung (Fig. 6K–M). Together, DSF dramatically impaired USP21-induced activation of the G3BP1/Wnt/β-Catenin signaling axis and inhibited USP21-driving ESCC progression.

### The clinical significance of the USP21/G3BP1 axis in ESCC patients

**Fig. 7:**
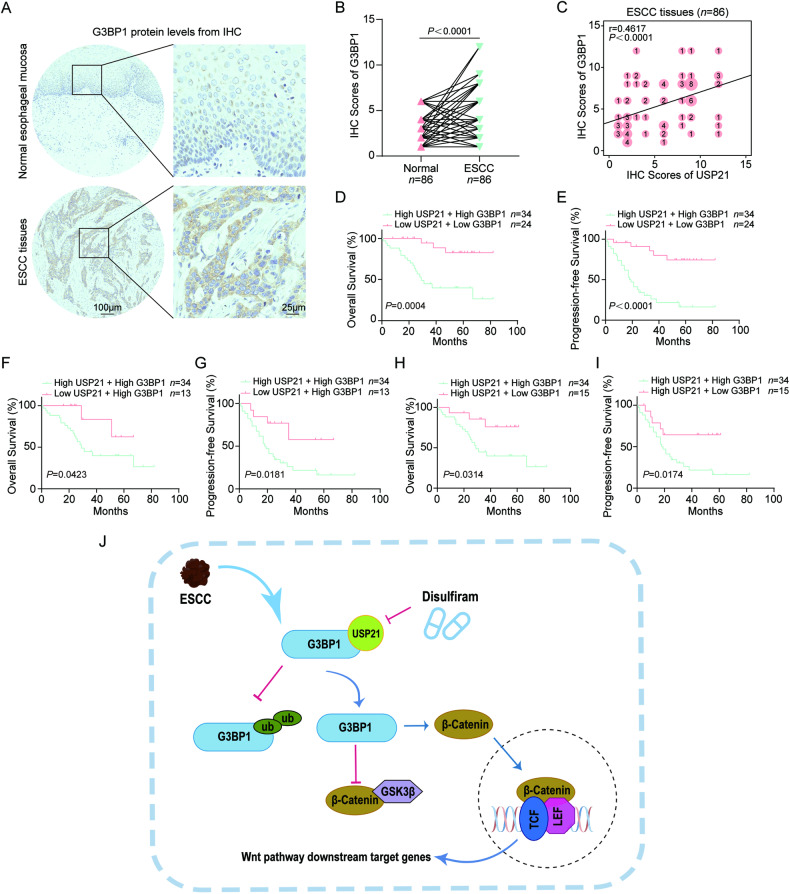
The clinical significance of the USP21/G3BP1 axis in ESCC patients. **A**, **B** G3BP1 protein expression was determined using IHC in the ESCC samples (86 pairs). Representative IHC staining of G3BP1 with scale bars (**A**) and quantification for IHC scores (**B**) as displayed. **C** Spearman correlation analysis was performed to evaluate the association between USP21 and G3BP1 protein levels in 86 pairs of ESCC tumor tissues. Kaplan-Meier curves with log-rank test were conducted to evaluate OS and PFS rates between ESCC patients with simultaneously high USP21 and G3BP1 expression and those with low protein levels of USP21 and G3BP1 (**D**, **E**), those with low USP21 and high G3BP1 protein levels (**F**, **G**), or those with high USP21 and low G3BP1 expression (**H**, **I**). **J** The schematic diagram was created by Figdraw, which illuminates the regulatory mechanism of the USP21/G3BP1/Wnt/β-Catenin axis in ESCC malignant progression. The Wilcoxon matched-pairs signed rank test was performed in (**B**) while a log-rank test was used in (**D**–**I**). All *P* values and n-numbers as shown.

To explore the potential function of the USP21/G3BP1 axis in ESCC patients, we continued to evaluate G3BP1 protein levels in 86 pairs of ESCC tissues. IHC scores exhibited that G3BP1 protein had an obvious increase in ESCC tumor samples compared to that in normal esophageal mucosa (Fig. 7A, B). ROC analysis suggested that G3BP1 protein levels have the potential ability to distinguish normal and ESCC tissues (Fig. S11A). Similar to USP21, higher protein levels of G3BP1 were found in ESCC tissues with T3-4 or N1-3 when compared to tumor samples with T1-2 (Fig. S11B) or N0 (Fig. S11C). ESCC samples with more advanced stages (III or IV) had higher G3BP1 protein levels than those with stage I or II (Fig. S11D). ESCC tissues in Stage II also exhibited higher levels of G3BP1 protein than those in Stage I (Fig. S11D). In addition, ESCC samples with poor differentiation (G3) showed upregulated G3BP1 protein levels compared to tumor tissues with well/moderate differentiation (G1-2) (Fig. S11E). Nonetheless, there was no significant correlation between other clinical factors (age and gender) and G3BP1 protein expression (Fig. S11F, G). Additionally, survival analyses revealed a strong correlation between G3BP1 protein levels and OS/PFS (Fig. S11H, I). More importantly, spearman correlation analysis showed that increased USP21 protein levels were positively correlated to upregulated protein expression of G3BP1 (Fig. 7C). ESCC patients with simultaneously high protein levels of USP21 and G3BP1 showed a worse OS and PFS than those with low expression of USP21 and G3BP1 (Fig. 7D, E), with low USP21 and high G3BP1 expression (Fig. 7F, G), or with high USP21 and low G3BP1 expression (Fig. 7H, I). Together, these results suggest aberrant activation of the USP21/G3BP1 axis in ESCC, which indicates an unfavorable clinical outcome.

## Discussion

ESCC is one of the most common human malignancies in the Asian population, however, effective therapeutic targets are still severely lacking [39–41]. In the present study, we demonstrated that USP21 plays an oncogenic role in ESCC progression, which is dependent on its activity of deubiquitination. Mechanistically, USP21 physically interacted with G3BP1 to enhance the protein stability of the latter. Furthermore, USP21-mediated accumulation of G3BP1 protein activates the Wnt/β-Catenin pathway, which promotes ESCC malignant progression (Fig. 7J).

In recent few years, increasing evidence has verified that USP21 functions as an oncogene in a variety of human cancers, including gastric cancer, pancreatic cancer, liver cancer, renal cell carcinoma, colorectal cancer, breast cancer, and lung cancer [15, 17–20, 23–25]. Among these reports, the oncogenic role of USP21 almost all depends on its deubiquitinase. For instance, USP21 induced the activation of the ERK signaling pathway by deubiquitinating MEK2 and stabilizing it, which promoted the growth of hepatocellular cancer [19]. Liu et al. reported that USP21 enhanced the ability of tumor cells to repair DNA damage by interacting, deubiquitinating, and stabilizing BRCA2 in hepatocellular cancer [20]. Another study about basal-like breast cancer reported that USP21 promoted the progression of the cell cycle and resistance to paclitaxel by regulating FOXM1 deubiquitination [15]. According to the study by Chen et al., USP21 increased the stabilization of EZH2 to promote cell proliferation and metastasis in bladder cancer [14]. In human pancreatic ductal adenocarcinoma, USP21 activated the Wnt pathway to promote the stemness of cancer cells by acting as a deubiquitinase of TCF7 [24]. Yun et al. reported that USP21 promoted the liver metastasis of colorectal cancer cells via deubiquitinating and stabilizing Fra-1 [17]. A study in lung cancer revealed that USP21 was upregulated in non-small cell lung cancer and promoted cell proliferation, migration, and invasion by stabilizing Yin Ynag-1 (YY1), a well-known oncogene, which further increased USP21 expression level via the positive feedback regulation of YY1/SNHG16/miR-4500 axis [23]. In gastric cancer, USP21 increased the expression level of MAPK1 to promote malignant progression by binding and stabilizing the transcription factor GATA3 [18]. Zhou et al. demonstrated that the deubiquitinase activity of USP21 was required for its oncogenic role in cholangiocarcinoma, although no direct targets have been explored [42]. Collectively, all these reports suggested that the abnormal deubiquitinase of USP21 was crucial for its tumor-promoting function in most human cancers. Interestingly, one study reported that USP21 promoted the tumorigenic properties by acting as a transcription factor to initiate the transcription of IL-8 in renal cell carcinoma which indicates the deubiquitinase-independently cancer-promoting mechanism of USP21 [25].

Upon preparing this manuscript, one recent study reported that USP21 promotes proliferation and glycolysis of esophageal cancer cells [30], however, whether the oncogenic role of USP21 depends on its deubiquitinase activity or not remains unknown. In the present study, we first demonstrated USP21 functions as an oncogene to accelerate ESCC proliferation and metastasis in a deubiquitinase-dependent manner. Importantly, we identified G3BP1 as the direct target of USP21 and investigated that USP21-regulated deubiquitination and stabilization of G3BP1 were required for the activation of Wnt/β-Catenin signaling and ESCC progression. Additionally, our study, for the first time, identifies USP21 as a prognostic predictor for ESCC patients and determines the differences in USP21 expression in different stages of ESCC. It is worth noting that the multivariate analysis based on Cox-regression Hazard models revealed that USP21 protein expression (High vs. Low) can independently predict PFS rather than OS, while the status of regional lymph node (N1-3 vs. N0) is an independent predictor for both PFS and OS. This suggests that USP21 may be no more effective than standard staging in the prognostic prediction of ESCC. Another issue that needs to be clarified is that although USP21 protein levels could distinguish ESCC tumors from their matched normal tissues, its diagnostic value for ESCC should be validated using the standard approach which would require matched biopsies from non-cancer patients.

Contrary to the cancer-promoting function of USP21, Nguyen et al. found that ectopic expression of USP21^WT^ suppressed the activity of YAP by stabilizing MARK kinases, while USP21 depletion exhibited an enhanced effect on YAP activity [43]. In addition, knocking down USP21 promoted the growth of A549 (lung cancer cells) and MDA-MD-231 (breast cancer cells) in soft agar which suggests the tumor-suppressing role of USP21 [43]. Considering the oncogenic role of USP21 in the reports from Peng et al., Arceci et al., and Xu et al. [15, 16, 23], the potential function of USP21 in lung cancer and breast cancer is controversial, which is worth exploring further.

To dissect the oncogenic mechanisms led by USP21 in regulating ESCC progression in this study, Co-IP assays and mass spectrometry analysis were performed to obtain potential proteins binding to USP21, among which G3BP1 was selected for further exploration due to its relatively high enrichment in the Co-IP complex and oncogenic role in ESCC progression [33]. After verifying the physical interaction between G3BP1 and USP21 in KYSE-150 and Eca-109 cells, the essential domains for their binding were further mapped. Then, we found that USP21 positively regulated the G3BP1 level by decreasing its ubiquitination. To ask whether G3BP1 is involved in USP21-mediated malignance in ESCC progression, a series of rescue experiments were applied. We demonstrated that USP21-mediated enhancement of cell growth, migration, and invasion was remarkedly reversed by knocking down G3BP1 expression, while impaired malignant behaviors induced by USP21 depletion were obviously restored by ectopic expression of G3BP1. These results strongly supported the crucial role of G3BP1 in USP21-mediated ESCC proliferation and metastasis. As far as we know, G3BP1 is the first reported direct target for the USP21-drived ESCC progression.

It has been reported that G3BP1 participates in the regulation of the Wnt/β-Catenin signaling pathway [33–35]. Interestingly, one recent study from our lab also demonstrated the important role of the abnormally activated Wnt signaling pathway in ESCC proliferation and metastasis [44]. Therefore, we continued to explore the effects of USP21 on Wnt/β-Catenin signaling and found that USP21 positively mediated the Wnt/β-Catenin pathway in a G3BP1-dependent manner. To identify the essential role of Wnt/β-Catenin pathway in USP21/G3BP1 axis-regulated ESCC progression, IWR-1, an inhibitor for Wnt/β-Catenin signaling, was applied to treat KYSE-150 cells with USP21 overexpression, while laduviglusib, an activator for Wnt/β-Catenin signaling, was added into culture medium of KYSE-150 cells with USP21 depletion. Our results suggest that the USP21/G3BP1 axis-driving progression of ESCC is dependent on the activity of Wnt/β-Catenin signaling. Consistently, it has been demonstrated that dysregulated Wnt/β-Catenin signaling modulates cancer stem cells, DNA damage response, epithelial-mesenchymal transition, and metabolic reprogramming, which contributes to ESCC initiation, progression, and chemoradiotherapy resistance [45, 46]. Our study clarified the upstream regulatory mechanisms underlying abnormal activation of Wnt/β-Catenin signaling which provides new insight for the therapeutic strategy against this pathway.

Furthermore, we introduced DSF, against USP21 deubiquitylation activity, and explored its effect on USP21-mediated proliferation and metastasis in ESCC. Our results demonstrated that DSF treatment impaired USP21-mediated upregulation of G3BP1, activation of Wnt/β-Catenin signaling, and acceleration of ESCC progression, which further supports the crucial role of deubiquitylation activity on USP21-mediated oncogenesis in ECSS. Finally, we explored the association between the expression of USP21 and G3BP1 proteins in 86 pairs of ESCC samples. A series of analyses based on IHC scores indicates that the regulatory axis of USP21/G3BP1 definitely exists in ESCC and its dysregulation predicts advanced stages and worse clinical prognosis.

Collectively, our study manifested the tumor-promoting regulation of the USP21/G3BP1 axis in ESCC progression. Considering the urgent shortage of potent targets for ESCC therapy, our results provided a promising insight and an important theoretical basis to develop the therapeutic strategy for ESCC.

### Supplementary information

Supplementary Information

## Data Availability

All data supporting the results of this study are included within this article, its supplementary files, and original data.
