# Supplementary material for: USP21-mediated G3BP1 stabilization accelerates proliferation and metastasis of esophageal squamous cell carcinoma via activating Wnt/β-Catenin signaling
Source: Oncogenesis. 2024 Jun 21;13(1):23. doi: 10.1038/s41389-024-00524-3 (PMC11192907; doi:10.1038/s41389-024-00524-3)
Supplement: Supplementary file 1 — Supplementary Information [file 41389_2024_524_MOESM1_ESM.pdf]

Supplementary Information

USP21-mediated G3BP1 stabilization accelerates proliferation and metastasis of esophageal squamous cell carcinoma via activating Wnt/β-Catenin signaling

Jiazhong Guo, Yunpeng Zhao, et al.

***Corresponding author:**

Xiaogang Zhao, zhaoxiaogang@sdu.edu.cn

Peichao Li, lipeichao@email.sdu.edu.cn

Supplementary information included in this PDF file:

Supplementary Figures 1 to 11

Supplementary Tables 1 to 6

Fig. S1

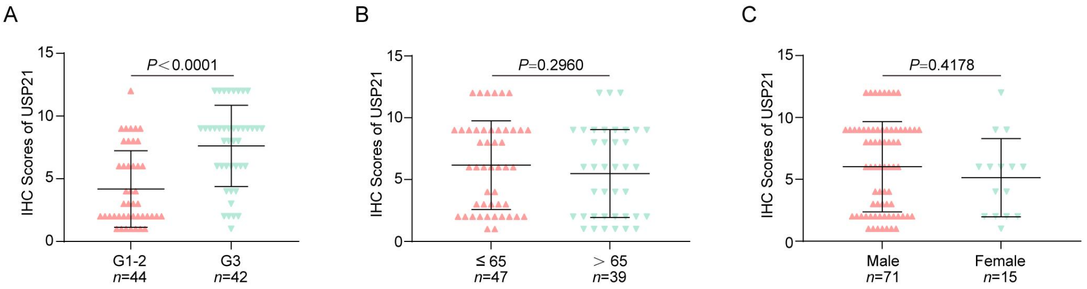

Fig. S1. USP21 protein levels in ESCC tumors with different differentiation, age, or gender. **A-C** The difference in USP21 IHC scores between different groups of differentiation (**A**), age (**B**), or gender (**C**). The Mann-Whitney test was used to evaluate statistical significance (**A-C**).

Fig. S2

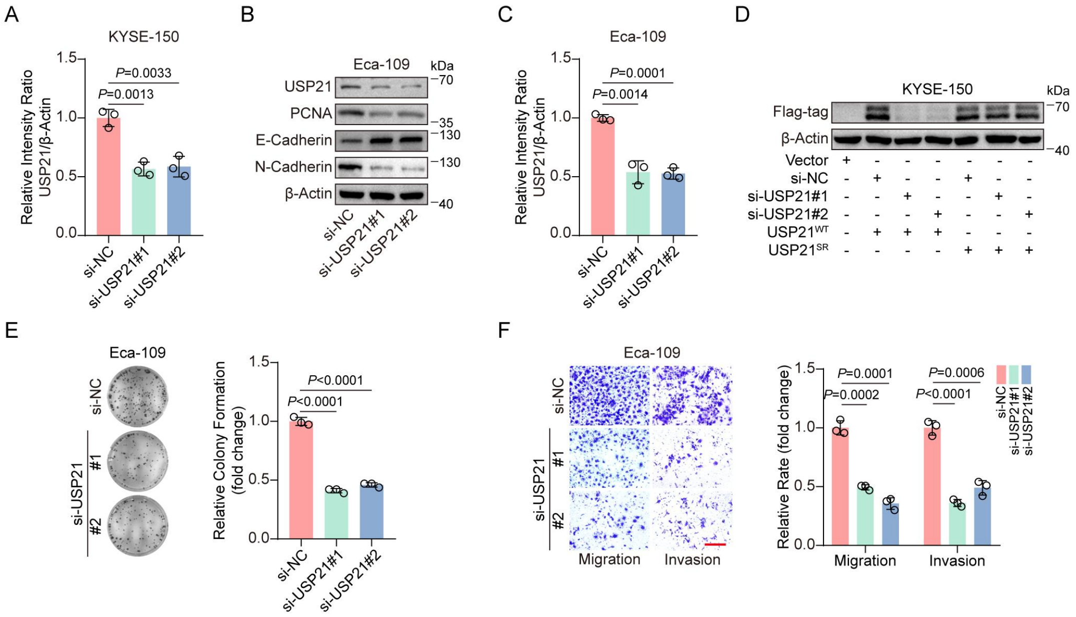

Fig. S2. USP21 depletion inhibits the growth, migration, and invasion of ESCC cells.

A The quantitation of western blots for the efficiency of USP21 depletion in KYSE-150 cells was performed. **B, C** Western blot analysis (**B**) and its quantitation (**C**) were applied to detect the levels of indicated proteins in Eca-109 cells with transfection of si-NC, si-USP21#1, or si-USP21#2. **D** KYSE-150 cells expressing Vector, USP21^{SR}, or USP21^{WT} were transfected with si-NC, si-USP21#1, or si-USP21#2, followed by western blot for Flag-tag. **E, F** Plate colony formation (**E**) and transwell assays (**F**) were used to evaluate the growth, migration, and invasion capability of Eca-109 cells with indicated transfection. Representative stainings and statistical quantification as indicated. The scale bar (red line) in (**F**) is 100 μm. The data are presented as means ± SD, and an unpaired *t*-test was used to identify the statistical significance in (**A, C, E, F**).

Fig. S3

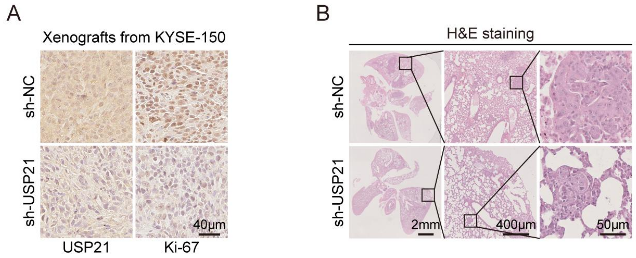

Fig. S3. IHC for USP21 and Ki-67 proteins and H&E staining for pulmonary metastatic nodules in xenograft models derived from KYSE-150 cells expressing sh-NC or sh-USP21.

A The IHC was performed to detect USP21 and Ki-67 protein levels in the sections of tumor tissues from mice xenografted with KYSE-150 cells expressing sh-NC or sh-USP21. **B** Representative images of H&E staining of metastatic nodules in mice lung from sh-NC or sh-USP21 group.

Fig. S4

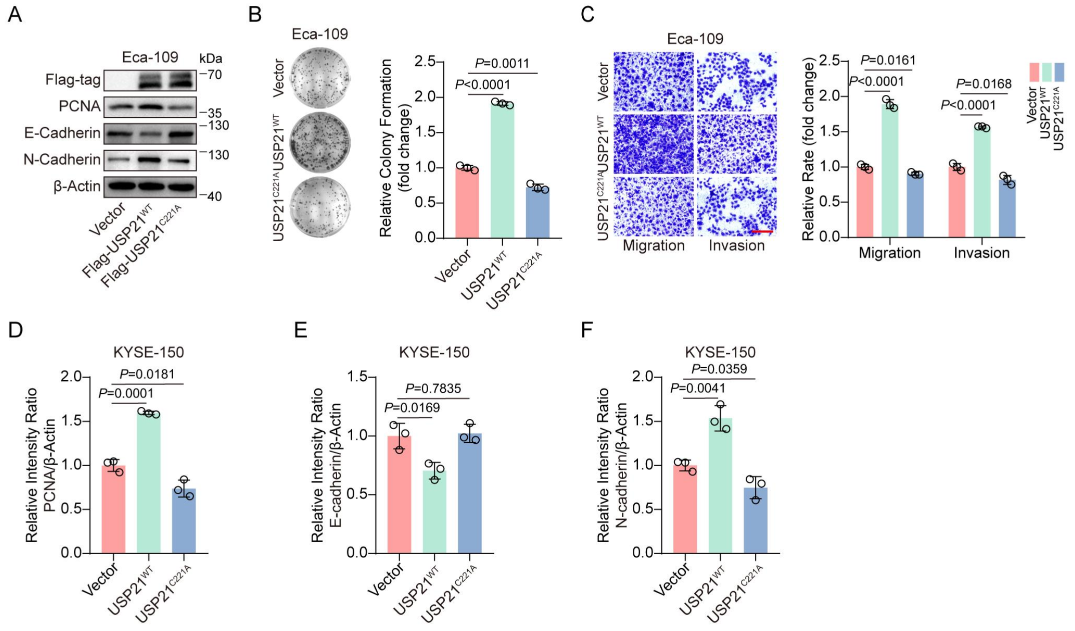

Fig. S4. USP21 functions as an oncogene in ESCC cells through its deubiquitinase activity.

A The expression of indicated proteins was detected by western blot in Eca-109 cells transfected with Vector, USP21^{WT}, or USP21^{C221A}. **B**, **C** Plate colony formation (**B)** and transwell assays (**C**) were performed to assess the growth, migration, and invasion of the Vector-, USP21^{WT}-, or USP21^{C221A}-expressing Eca-109 cells. Representative stainings and statistical analysis as shown. The scale bar (red line) in (**C**) is 100 µm. **D-F** The blots for PCNA (**D**), E-Cadherin (**E**), and N-Cadherin (**F**) in KYSE-150 cells expressing Vector, USP21^{WT}, or USP21^{C221A} were qualified. The data are displayed as means ± SD, and the statistical significance was identified using an unpaired *t*-test (**B-F**).

Fig. S5

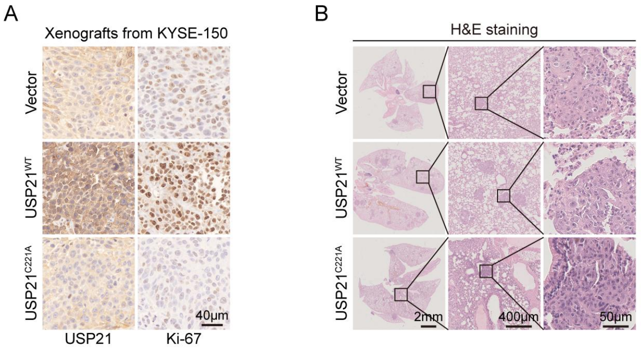

Fig. S5. IHC analysis of USP21 and Ki-67 proteins and H&E staining of pulmonary metastatic nodules in xenograft models derived from KYSE-150 cells with the expression of Vector, USP21^{WT}, or USP21^{C221A}.

A The protein levels of USP21 and Ki-67 were detected in tumor sections of subcutaneous xenograft mice models with KYSE-150 expressing Vector, USP21^{WT}, or USP21^{C221A}. **B** Representative H&E staining images from pulmonary metastatic nodules of mice injected with Vector-, USP21^{WT}-, or USP21^{C221A}-expressing KYSE-150 cells. Scale bars as shown on their respective panels.

.

Fig. S6

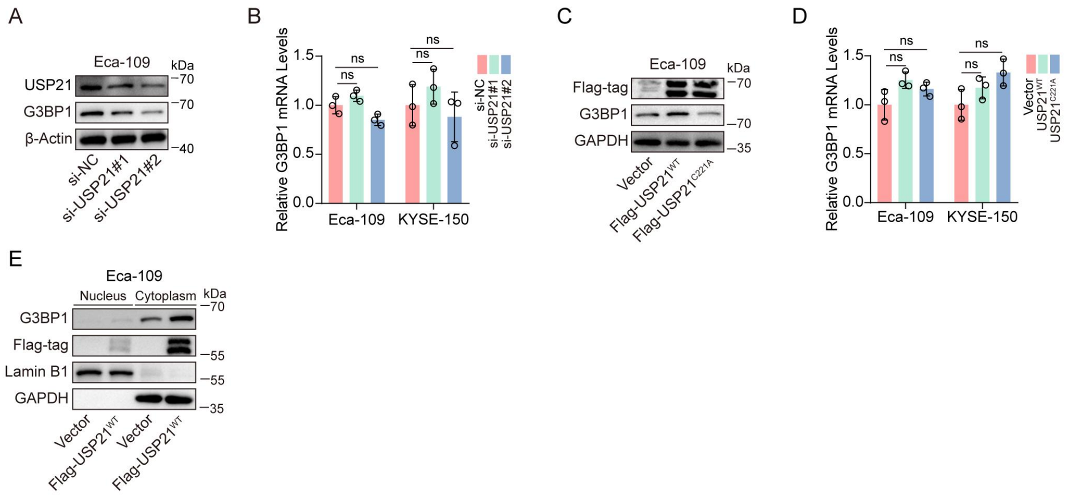

Fig. S6. USP21 depletion or overexpression alters G3BP1 protein levels but does not affect its mRNA expression.

A Western blot analysis was applied to determine the expression of G3BP1 protein in Eca-109 cells transfected with si-NC, si-USP21#1, or si-USP21#2. **B** G3BP1 mRNA levels were evaluated in KYSE-150 and Eca-109 cells with depletion of USP21 via RT-qPCR. **C** G3BP1 protein levels were assessed using a western blot in Eca-109 with ectopic expression of Vector, USP21^{WT}, or USP21^{C221A}. **D** RT-qPCR was conducted to measure G3BP1 mRNA levels in KYSE-150 and Eca-109 cells with USP21 overexpression. **E** Protein lysate was respectively collected from the cytoplasm or nucleus in Eca-109 cells expressing ectopic USP21^{WT} and then subjected to western blot for indicated proteins. GAPDH and Lamin B1 were used as an internal control for the cytoplasmic and nucleus protein lysate, respectively. Data are shown as mean values ± SD. An unpaired *t*-test was applied for the determination of statistical significance in (**B, D**). Corresponding *P*-values are indicated on the graphs.

Fig. S7

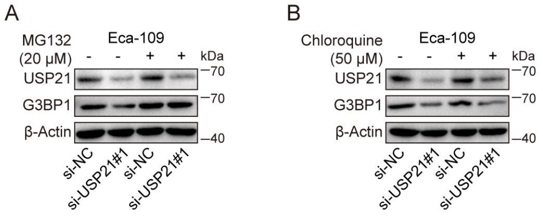

Fig. S7. MG132, not chloroquine, diminishes USP21-mediated upregulation of G3BP1 protein.

A, B Eca-109 cells with USP21 depletion were treated with MG132 (20 μM) **(A)** or chloroquine (50 μM) **(B)** for 24 hours followed by western blot analysis for G3BP1 proteins.

Fig. S8

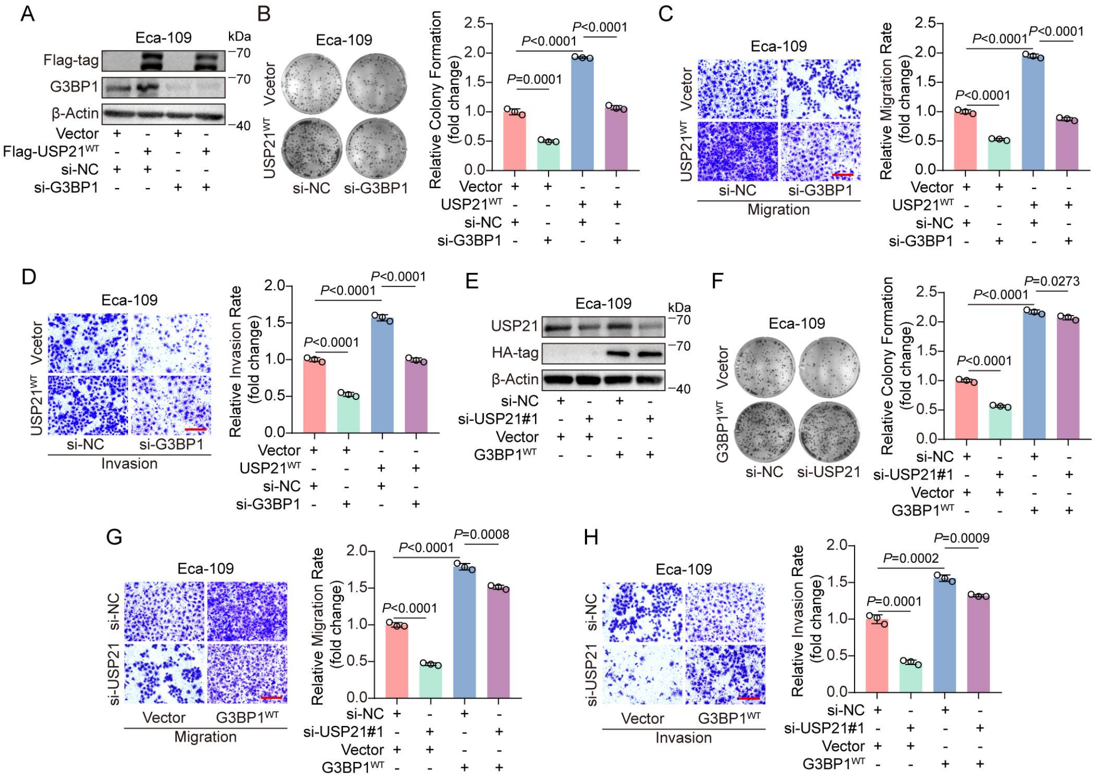

Fig. S8. G3BP1 is essential for USP21-mediated ESCC progression.

A Western blot analysis was used to detect indicated protein levels in Vector- or USP21^{WT}-expressing Eca-109 cells transfected with si-NC or si-G3BP1. **B-D** Plate colony formation (**B**) and transwell assays (**C**, **D**) were applied to evaluate the growth, migration, and invasion of Eca-109 cells with the expression of Vector + si-NC, USP21^{WT} + si-NC, Vector + si-G3BP1, or USP21^{WT} + si-G3BP1. Representative staining and statistical quantification as shown. **E** Western blot analysis was performed to determine indicated protein levels in Vector- or G3BP1^{WT}-expressing Eca-109 cells transfected with si-NC or si-USP21#1. **F-H** Plate colony formation (**F**) and transwell assays (**G**, **H**) were performed to assess the growth, migration, and invasion of Eca-109 cells expressing si-NC + Vector, si-NC + G3BP1^{WT}, si-USP21#1 + Vector, or si-USP21#1 + G3BP1^{WT}. Representative images and statistical quantification as indicated. Scale bars (red line) are 100 μm in (**C, D, G, H**). The data are presented as means ± SD and the unpaired *t*-test was used to determine the statistical significance (**B-D, F-H**). All *P*-values are indicated on the corresponding graphs.

Fig. S9

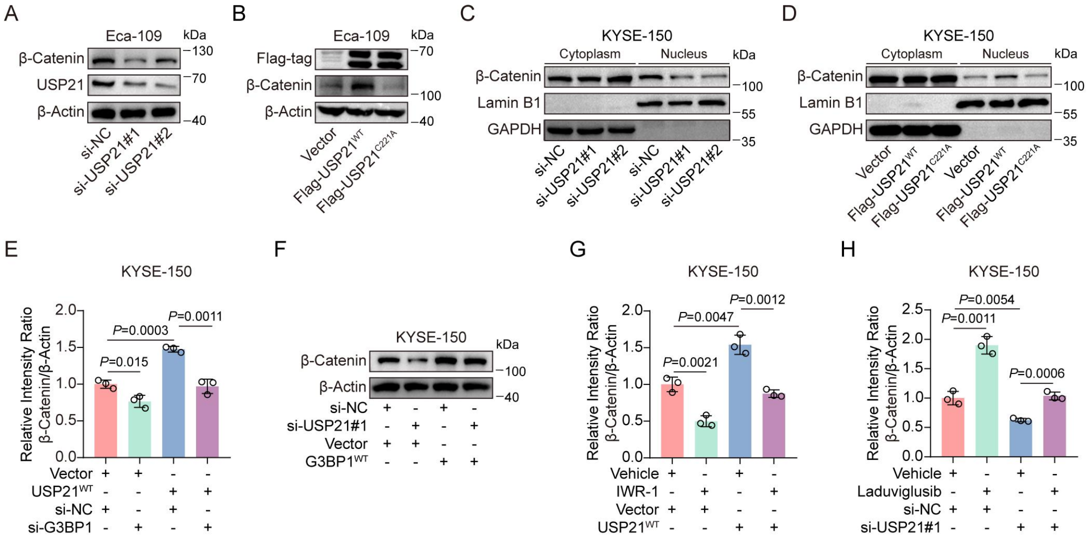

Fig. S9. USP21 regulates β-Catenin protein levels through G3BP1.

A, B The protein levels of β-Catenin were detected using western blot in Eca-109 cells with USP21 knockdown (**A**) or overexpression (**B**). **C, D** The cytoplasmic and nuclear proteins were extracted separately from KYSE-150 transfected with si-NC, si-USP21#1 or si-USP21#2 (**C**) or expressing Vector, USP21^{WT}, or USP21^{C221A} (**D**), followed by western blot detecting β-Catenin levels. **E** The quantitation of western blots was from three replicate experiments with indicated information. **F** KYSE-150 cells were transfected with si-NC + Vector, si-NC + G3BP1^{WT}, si-USP21#1+Vector, or si-USP21#1+G3BP1^{WT} followed by western blot for β-Catenin protein. **G, H** Western blots from indicated experiments repeated three times were quantified. The data are displayed as means ± SD. The statistical significance (**E, G, H**) was identified using the unpaired *t*-test, and all *P*-values are shown on the corresponding graphs.

Fig. S10

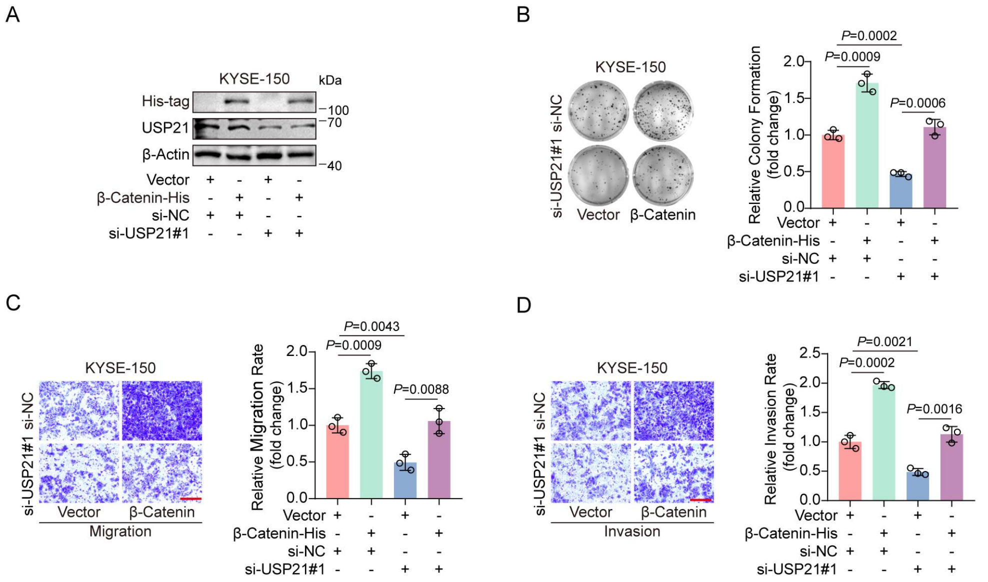

Fig. S10. Ectopic β-Catenin expression impairs si-USP21#1-induced inhibition on KYSE-150 cell proliferation, migration, and invasion.

A-D KYSE-150 cells were transfected with si-NC + Vector, si-NC + β-Catenin-His, si-USP21#1 + Vector, or si-USP21#1 + β-Catenin-His, followed by Western blot to detect β-Catenin protein (**A**), plate colony formation to assess cell growth (**B**), and transwell assays to evaluate cell migration and invasion (**C, D**). Representative stainings and quantification as indicated. Scale bars (red line) are 100 μm in (**C, D**). Statistical significance was verified using an unpaired *t*-test, and all analyzed data were shown as means ± SD (**C, D**). The *P*-values are respectively shown in the corresponding position.

Fig. S11

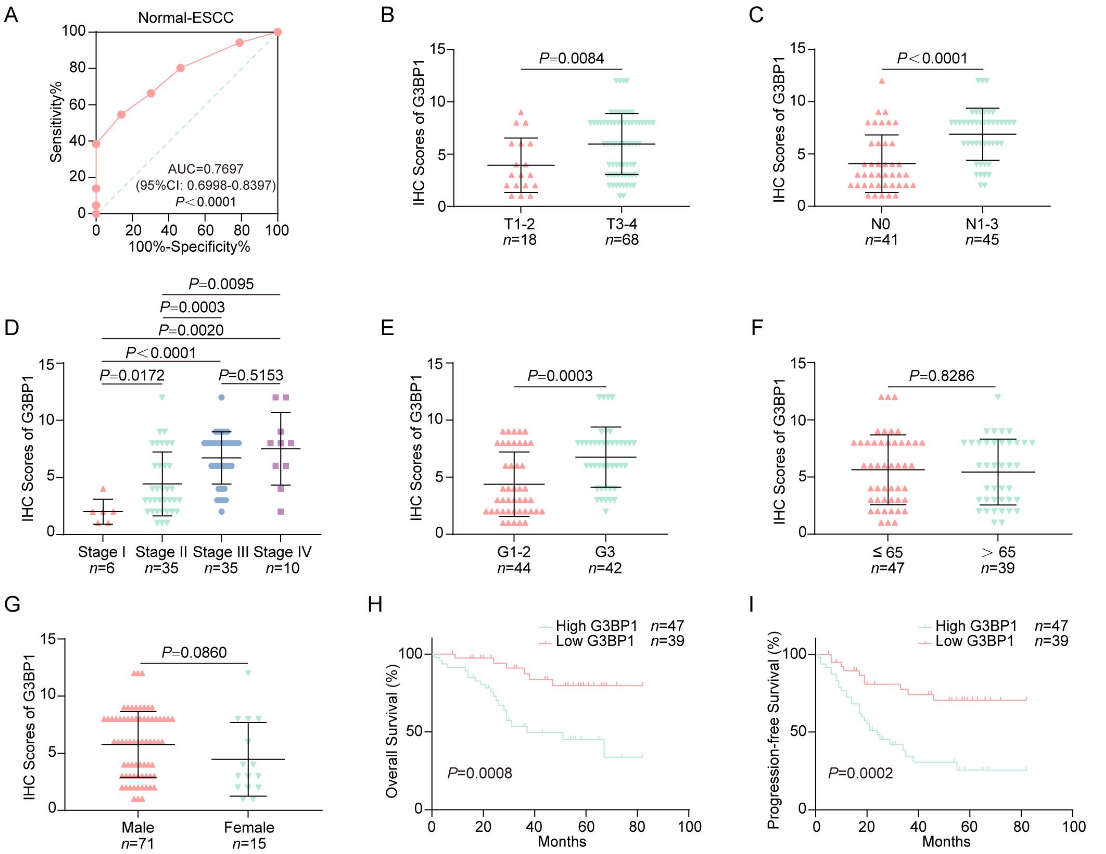

Fig. S11. The clinical significance of dysregulated G3BP1 protein expression.

A The ROC analysis was applied to assess the diagnostic value of G3BP1 protein expression for ESCC. **B-G** IHC scores of G3BP1 in ESCC tissues were compared among patients with different T statuses of primary tumor (**B**), with N0 or N1-3 statuses of regional lymph nodes (**C**), with different ESCC stages (**D**), or with different groups of differentiation (**E**), age (**F**), gender (**G**). **H, I** OS (**H**) and PFS (**I**) in ESCC patients with high or low G3BP1 protein levels were analyzed using Kaplan-Meier curves. The *P*-values were determined using a Mann-Whitney test in (**B-G**) and a log-rank test in (**H, I**). All *P*-values and n numbers as indicated.

Supplementary Table 1. Clinicopathologic factors from 86 ESCC patients.

Factors	No.
**Gender**	
Male	71
Female	15
**Age**	
≤ 65 years	47
> 65 years	39
**Primary Tumor**	
T1	6
T2	12
T3	66
T4	2
**Regional lymph node**	
N0	41
N1	17
N2	19
N3	9
**Metastasis**	
M0	86
M1	0
**TNM Stage**	
I	6
II	35
III	35
IV	10
**Differentiation**	
G1	18
G2	26
G3	42
**Prognostic follow-up**	
Available	86
Non-available	0

Supplementary Table 2. Detailed information of antibodies applied in this study.

**Antibody Names**	**Application**	**Source**
USP21 Antibody (B-9)	IHC	Santa Cruz Biotechnology (sc-515911)
USP21 Polyclonal antibody	WB, IP	Proteintech (17856-1-AP)
Anti-G3BP antibody	IHC, WB, IP	Abcam (ab181149)
β-Catenin Rabbit pAb	WB	Abclonal (A11932)
β-Catenin Rabbit mAb	IHC	Abclonal (A19657)
PCNA Rabbit mAb	WB	Abclonal (A12427)
Anti-DDDDK tag (Binds to FLAG® tag sequence) antibody	WB, IP	Abcam (ab205606)
HA tag Polyclonal antibody	WB, IP	Proteintech (51064-2-AP)
β-Actin Rabbit mAb (High Dilution)	WB	Abclonal (AC026)
GAPDH Monoclonal antibody	WB	Proteintech (60004-1-Ig)
Lamin B1 Polyclonal antibody	WB	Proteintech (12987-1-AP)
6*His, His-Tag Monoclonal antibody	WB	Proteintech (66005-1-Ig)
Anti-Ki67 antibody	IHC	Abcam (ab92742)
Rabbit IgG	IP	Beyotime (A7016)
Mouse IgG	IP	Beyotime (A7028)
Goat Anti-Mouse IgG（H+L）	WB	ZSGB-BIO(ZB-2305)
Goat Anti-Rabbit IgG（H+L）	WB	ZSGB-BIO(ZB-2301)
Mouse Anti-Rabbit IgG (Light-Chain Specific)	WB	CST (93702)

Supplementary Table 3. The information of plasmids used in this study.

**Plasmids names**	**Vector**	**Application**	**Source**
pLent-U6-GFP-Puro	pLent-U6-GFP-Puro	Vector for gene knockdown	WZ Biosciences Inc.
sh-USP21	pLent-U6-GFP-Puro	Knockdown of USP21	Tsingke Biotechnology
pLVX-puro	pLVX-puro	Vector for gene expression	Tsingke Biotechnology
Flag-USP21^{WT}	pLVX-puro	Ectopic gene expression	Tsingke Biotechnology
Flag-USP21^{C221A}	pLVX-puro	Ectopic gene expression	Tsingke Biotechnology
Flag-USP21-Δ1 (deletion: 134-152aa)	pLVX-puro	Ectopic gene expression	MiaoLing Biology
Flag-USP21-Δ2 (deletion: 212-558aa)	pLVX-puro	Ectopic gene expression	MiaoLing Biology
HA-G3BP1^{WT}	pLVX-puro	Ectopic gene expression	MiaoLing Biology
HA-G3BP1-Δ1 (deletion: 11-133aa)	pLVX-puro	Ectopic gene expression	MiaoLing Biology
HA-G3BP1-Δ2 (deletion: 340-415aa)	pLVX-puro	Ectopic gene expression	MiaoLing Biology
6xHis-Ubiquitin	pcDNA3.1	Polyubiquitination detection assay	MiaoLing Biology
pMD2.G	pMD2.G	Packaging of lentiviruses	Tsingke Biotechnology
psPAX2	psPAX2	Packaging of lentiviruses	Tsingke Biotechnology
TOPFlash	pGL6-TA	Dual-luciferase reporter assay	Beyotime
FOPFlash	pGL6-TA	Dual-luciferase reporter assay	Beyotime
pRL-TK	pRL-TK	Dual-luciferase reporter assay	Tsingke Biotechnology
Flag-USP21^{SR}	pLVX-puro	Ectopic gene expression	MiaoLing Biology
β-Catenin-His	pcDNA3.1	Ectopic gene expression	MiaoLing Biology

Supplementary Table 4. The sequence of siRNAs applied in the present study.

siRNA	Sequence (5'-3')	Application
si-USP21#1	Forward: GCAAGAUUGUGGACCUGUUTT	Knockdown
	Reverse: AACAGGUCCACAAUCUUGCTT	
si-USP21#2	Forward: CUGGUUGGCAUGUCUACAATT	Knockdown
	Reverse: UUGUAGACAUGCCAACCAGTT	
si-G3BP1	Forward: CAAGAUUCGCCAUGUUGAUTT	Knockdown
	Reverse: AUCAACAUGGCGAAUCUUGTT	

Supplementary Table 5. The sequence of primers used in this study.

**Targeted genes**	**Sequence (5'-3')**	**Application**
USP21	Forward: AGGTGTCTCTGCGGGATTGTT	qPCR
	Reverse: CGATTCAGATGGAGCACGAGG	
G3BP1	Forward: AGCCTGTTCAGAAAGTCCTTAGC	qPCR
	Reverse: CGAAGGCGATTATCTCGTCGGT	
ACTB	Forward: AGAGCCTCGCCTTTGCCG	qPCR
	Reverse: GAATCCTTCTGACCCATGCCC	
GAPDH	Forward: CATCACTGCCACCCAGAAGACTG	qPCR
	Reverse: ATGCCAGTGAGCTTCCCGTTCAG	

Supplementary Table 6. Multivariate analysis of prognostic factors for OS and PFS.

Variables	OS			PFS		
	HR	95% CI	*P*-value	HR	95% CI	*P*-value
**Gender**<br>Male vs. Female (Ref)	0.591	0.215-1.626	0.308	0.536	0.240-1.199	0.129
**Age**<br>> 65 vs. ≤ 65 years (Ref)	1.149	0.512-2.583	0.736	1.694	0.864-3.319	0.125
**Primary Tumor**<br>T3-4 vs. T1-2 (Ref)	3.463	0.693-17.297	0.130	1.561	0.607-4.014	0.356
**Regional lymph node**<br>N1-3 vs. N0 (Ref)	4.950	1.847-13.263	**0.001**	6.258	2.720-14.400	**0.000**
**Differentiation**<br>G3 vs G1-2 (Ref)	1.337	0.546-3.272	0.524	0.868	0.428-1.760	0.695
**USP21 protein levels**<br>High vs. Low (Ref)	2.233	0.768-6.495	0.140	2.563	1.121-5.861	**0.026**

OS, overall survival; PFS, progression-free survival; HR, hazard ratio; CI, confidence interval; Ref, Reference. The *P*-value was calculated by the Cox-regression Hazard models.
